# A New Late Miocene Odobenid (Mammalia: Carnivora) from Hokkaido, Japan Suggests Rapid Diversification of Basal Miocene Odobenids

**DOI:** 10.1371/journal.pone.0131856

**Published:** 2015-08-05

**Authors:** Yoshihiro Tanaka, Naoki Kohno

**Affiliations:** 1 Hokkaido University Museum, Kita 8 Nishi 5, Kita-ku, Sapporo, Hokkaido, 060–0808, Japan; 2 Department of Geology, University of Otago, 360 Leith walk, PO Box 56, Dunedin, 9054, New Zealand; 3 Numata Fossil Museum, 2-7-49, 1 Minami, Numata Town, Hokkaido, 078–2202, Japan; 4 Department of Geology and Paleontology, National Museum of Nature and Science, 4-1-1 Amakubo, Tsukuba, 305–0005, Japan; 5 Graduate School of Life and Environmental Sciences, University of Tsukuba, 1-1-1 Tennodai, Tsukuba, 305–8752, Japan; NYIT College of Osteopathic Medicine, UNITED STATES

## Abstract

The modern walrus, *Odobenus rosmarus*, is specialized and only extant member of the family Odobenidae. They were much more diversified in the past, and at least 16 genera and 20 species of fossil walruses have been known. Although their diversity increased in the late Miocene and Pliocene (around 8–2 Million years ago), older records are poorly known. A new genus and species of archaic odobenid, *Archaeodobenus akamatsui*, gen. et sp. nov. from the late Miocene (ca. 10.0–9.5 Ma) top of the Ichibangawa Formation, Hokkaido, northern Japan, suggests rapid diversification of basal Miocene walruses. *Archaeodobenus akamatsui* is the contemporaneous *Pseudotaria muramotoi* from the same formation, but they are distinguishable from each other in size and shape of the occipital condyle, foramen magnum and mastoid process of the cranium, and other postcranial features. Based on our phylogenetic analysis, *A*. *akamatsui* might have split from *P*. *muramotoi* at the late Miocene in the western North Pacific. This rapid diversification of the archaic odobenids occurred with a combination of marine regression and transgression, which provided geological isolation among the common ancestors of extinct odobenids.

## Introduction

**Fig 1:**
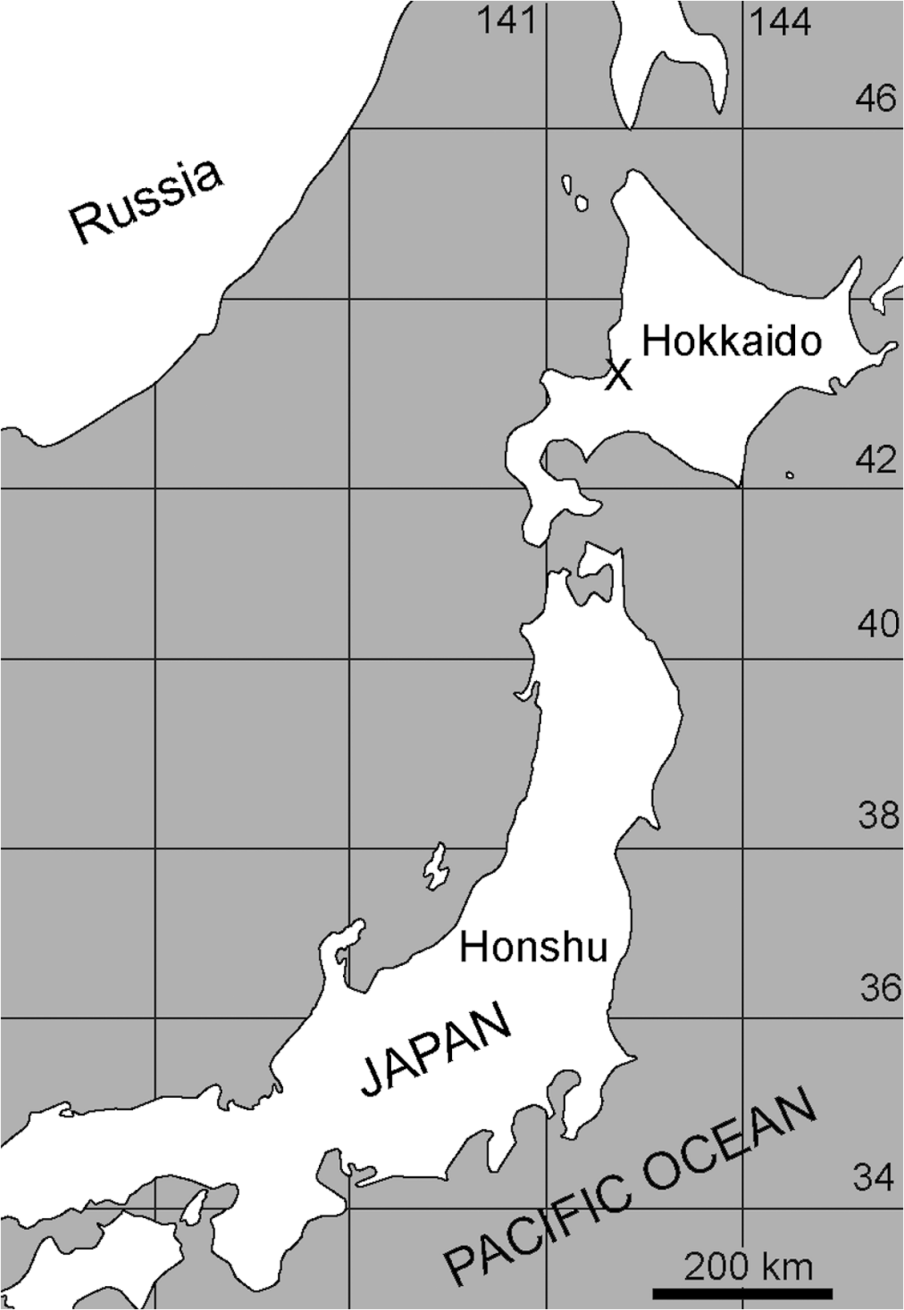
Map showing the holotype locality of *Archaeodobenus akamatsui*, gen. et sp. nov.

**Fig 2:**
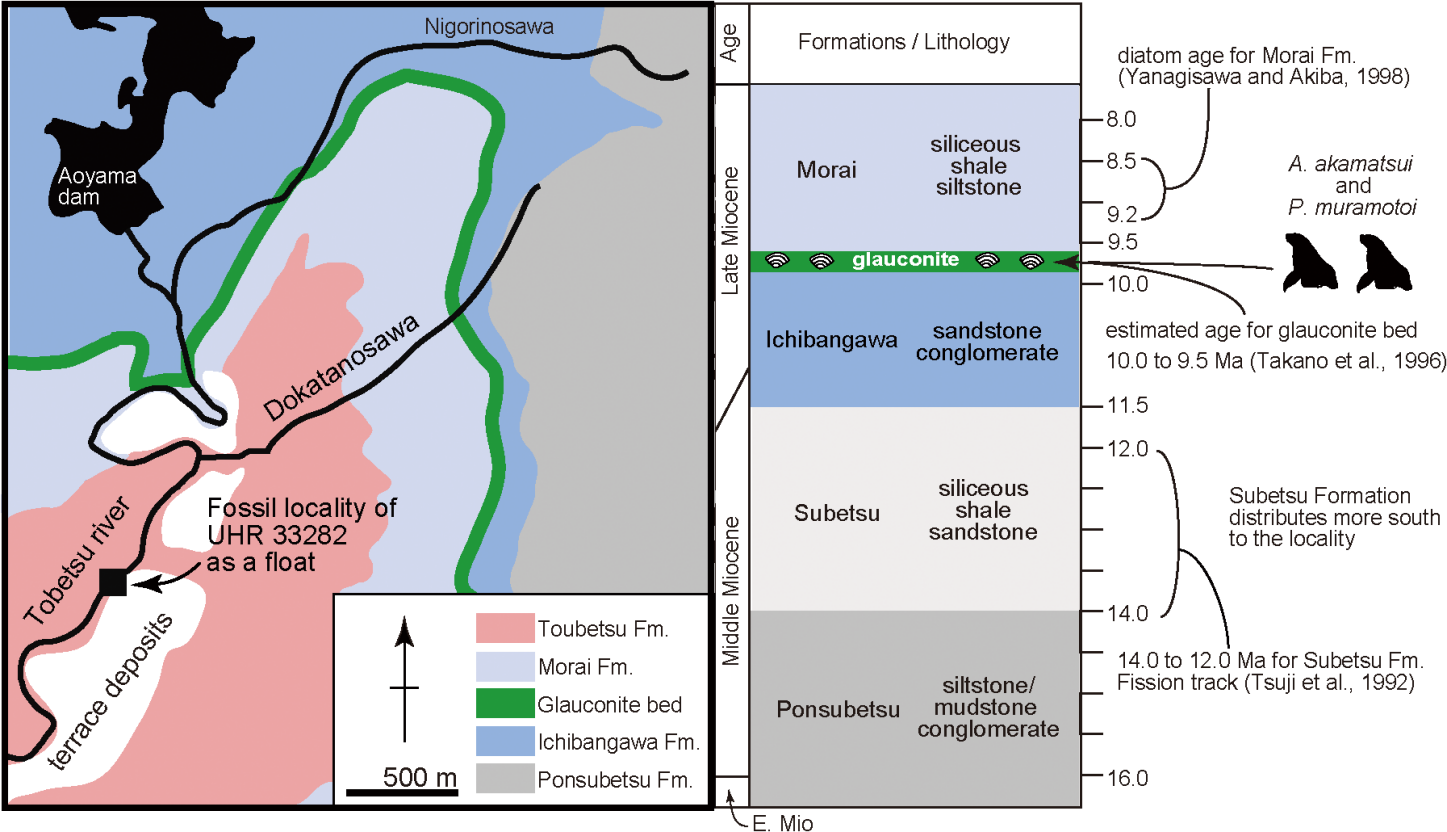
Locality map and stratigraphic sections of the *Archaeodobenus akamatsui* holotype locality, based on Takano et al. (1996).

A partial skeleton of a fossil pinniped was collected from the late Miocene Ichibangawa Formation in Tobetsu Town, Hokkaido, Japan (Figs 1 and 2). This skeleton includes a partial cranium, mandibles, anterior vertebrae and some appendicular bones. Previously, the holotype and paratype of the extinct odobenid, *Pseudotaria muramotoi* Kohno, 2006, were collected from the same formation in the same locality area. The new fossil has similar character combinations to those in *P*. *muramotoi*, suggesting that it also belongs to the family Odobenidae. However, the third specimen has some unique characters differentiation it from the previously known specimens of *P*. *muramotoi* from the same locality area and also other species in the Odobenidae. In the present paper, we describe the new fossil as a new genus and species of the family Odobenidae and examine its phylogenetic relationships among them to provide additional information and understand the evolutionary diversification of the odobenids during the Miocene.

### Ethics statement

No permits were required for the described study, which complied with all relevant regulations.

### Nomenclatural Acts

The electronic edition of this article conforms to the requirements of the amended International Code of Zoological Nomenclature, and hence the new names contained herein are available under that Code from the electronic edition of this article. This published work and the nomenclatural acts it contains have been registered in ZooBank, the online registration system for the ICZN. The ZooBank LSIDs (Life Science Identifiers) can be identified and the associated information viewed through any standard web browser by appending the LSID to the prefix "http://zoobank.org/". The LSID for this publication is: urn:lsid:zoobank.org:author:E500AA1E-C47F-48C5-9A9D-9B8C0959E8FE. The electronic edition of this work was published in a journal with an ISSN, and has been archived and is available from the following digital repositories: PubMed Central, LOCKSS.

### Systematic paleontology

MAMMALIA Linnaeus, 1758

CARNIVORA Bowdich, 1821

ODOBENIDAE Allen, 1880

*Archaeodobenus* gen. nov.

urn:lsid:zoobank.org:act:F509D05A-CE42-472F-9311-83405695F5F6

#### Type species

*Archaeodobenus akamatsui* sp. nov.

#### Diagnosis

As for the only species.

#### Etymology

The generic name, *Archaeodobenus*, is named in Greek archaios meaning ancient, and the type genus name of the family Odobenidae.

#### Distribution

Known from the late Miocene (ca. 10.0–9.5 Ma), Japan.

*Archaeodobenus akamatsui* sp. nov.

urn:lsid:zoobank.org:act:3ED6F1B3-24C7-4461-B32D-AE3096FB1C62

(Figs 3–10, Tables 1–3)

**Fig 3:**
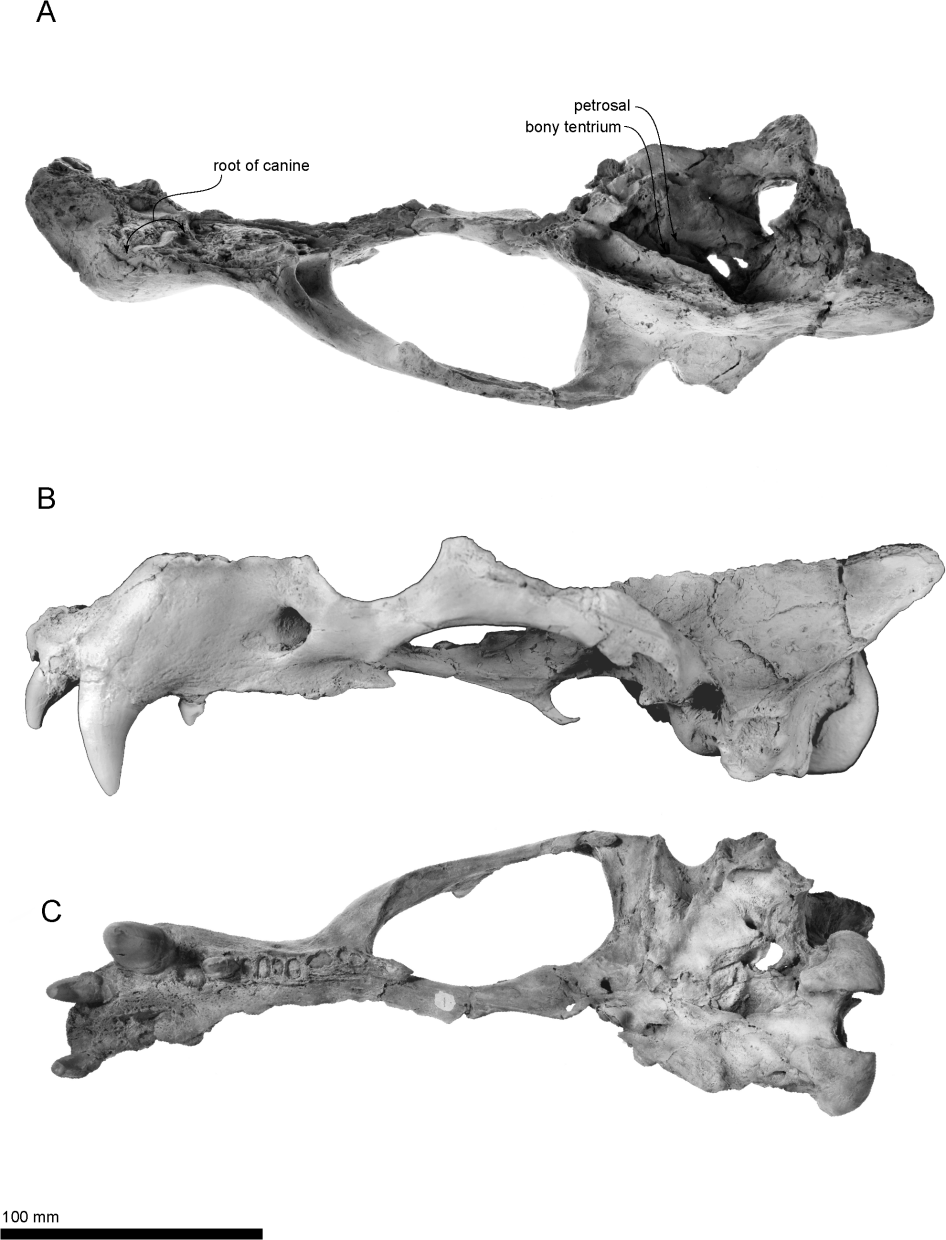
The holotype cranium of *Archaeodobenus akamatsui*. (A) dorsal view, (B) left lateral view, (C) ventral view.

**Fig 4:**
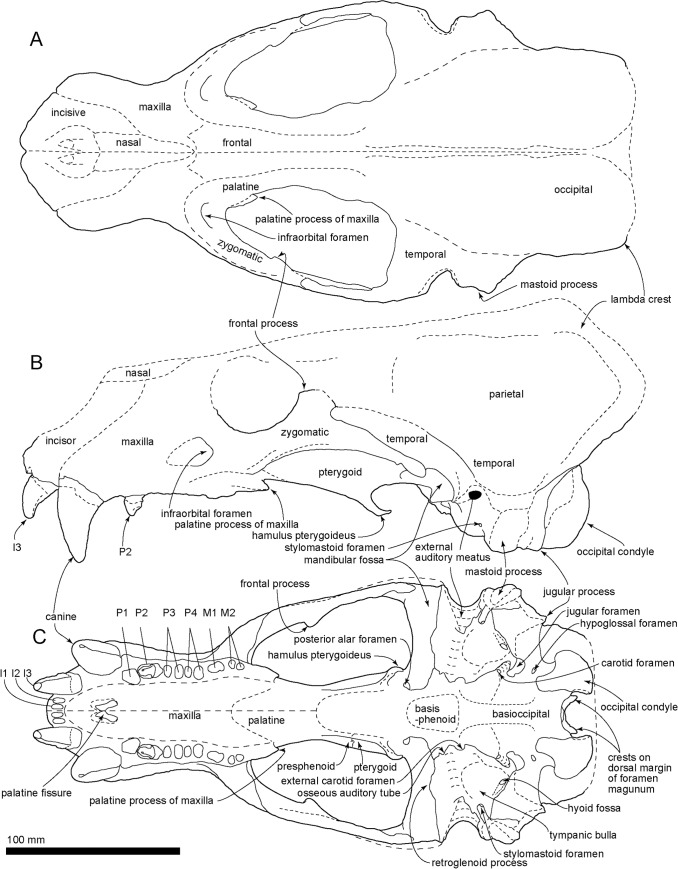
The reconstructed cranium of *Archaeodobenus akamatsui*. (A) dorsal view, (B) left lateral view, (C) ventral view.

**Fig 5:**
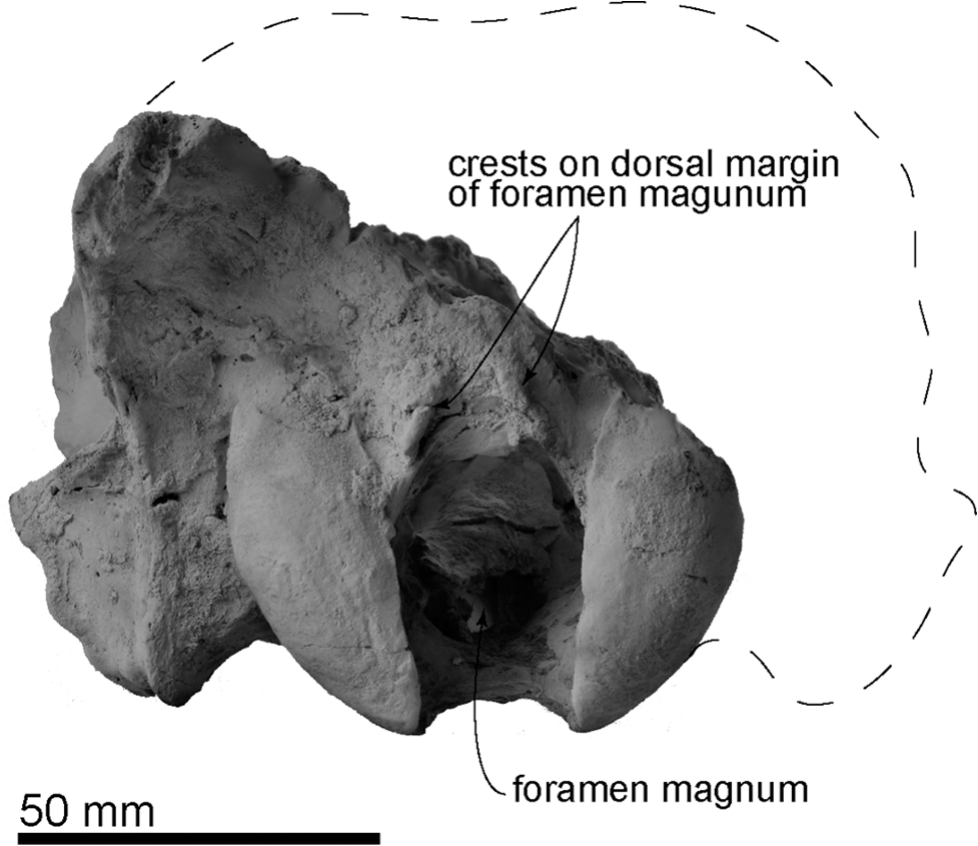
The holotype cranium of *Archaeodobenus akamatsui* in posterior view.

**Fig 6:**
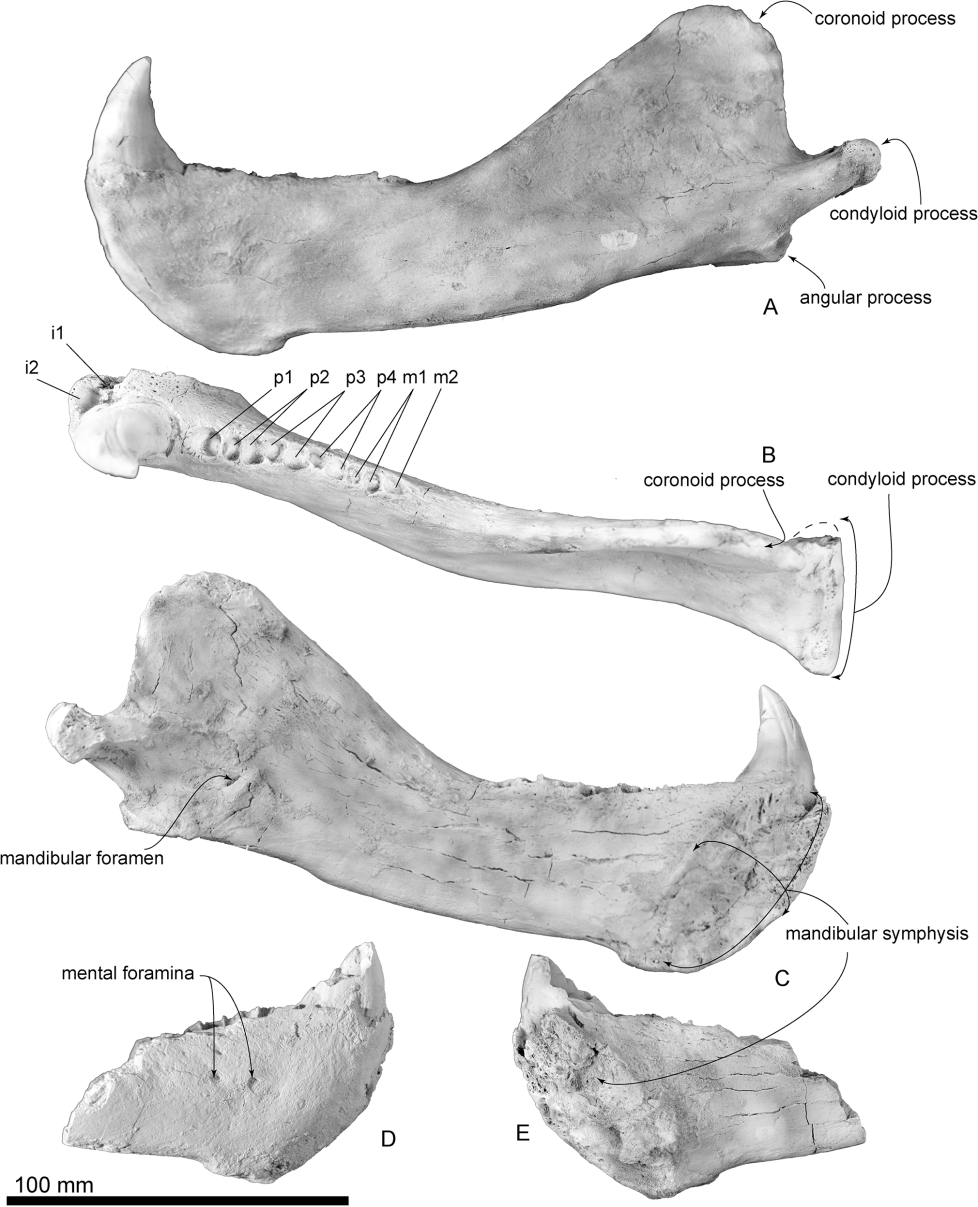
The holotype mandibles of *Archaeodobenus akamatsui*. (A)—(C) left mandible, (D)—(E) right mandible, (A) lateral view, (B) dorsal view, (C) medial view, (D) lateral view, (E) medial view.

**Fig 7:**
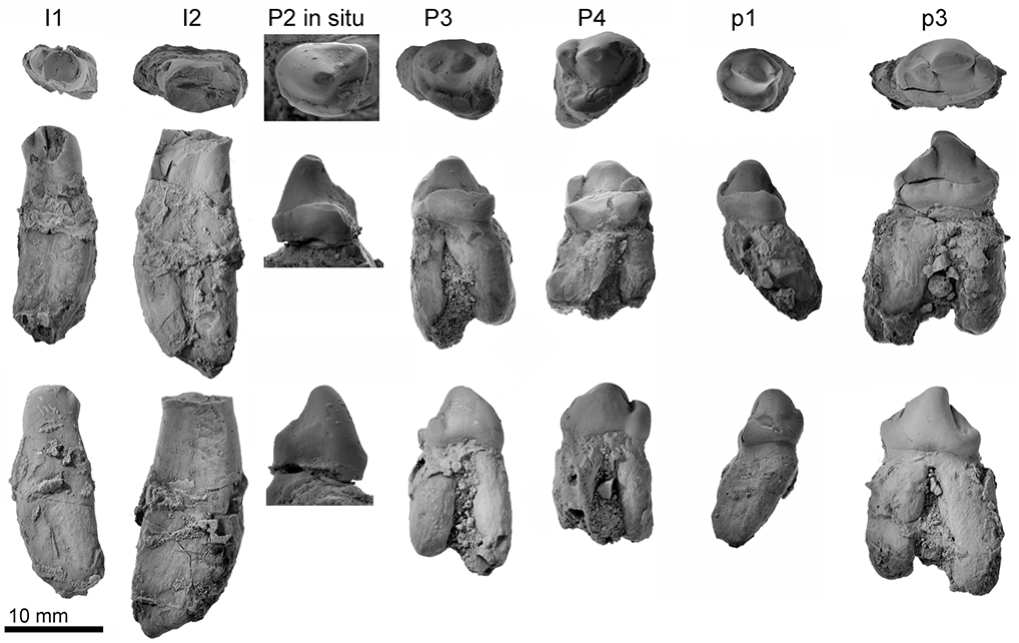
Photographs of the holotype teeth of *Archaeodobenus akamatsui*, in top row, occlusal; middle row, lingual; bottom row, buccal aspects.

**Fig 8:**
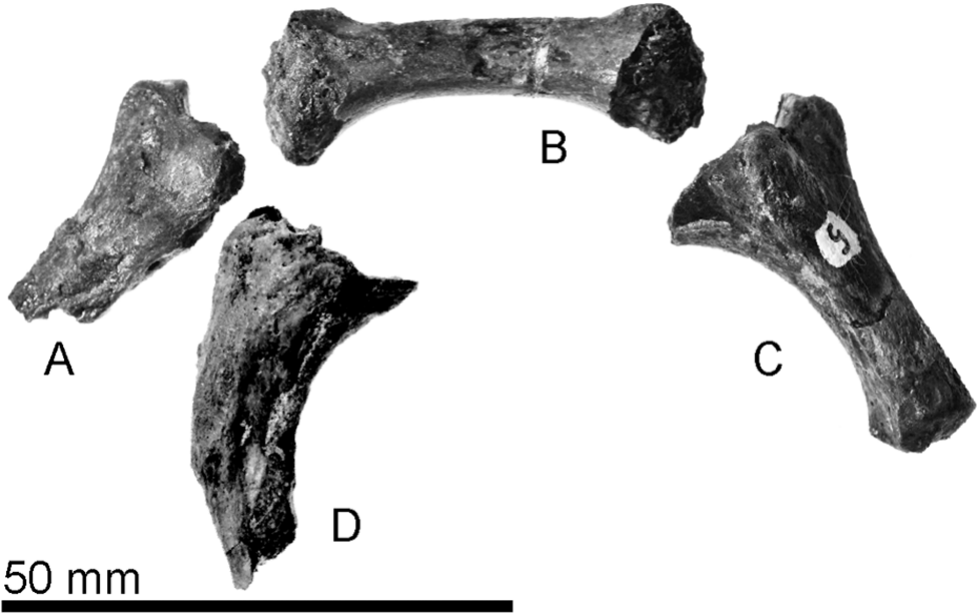
The holotype hyoids of *Archaeodobenus akamatsui*. (A) left ceratohyoid, (B) basihyoid, (C) right ceratohyoid, (D) left thyrohyoid.

**Fig 9:**
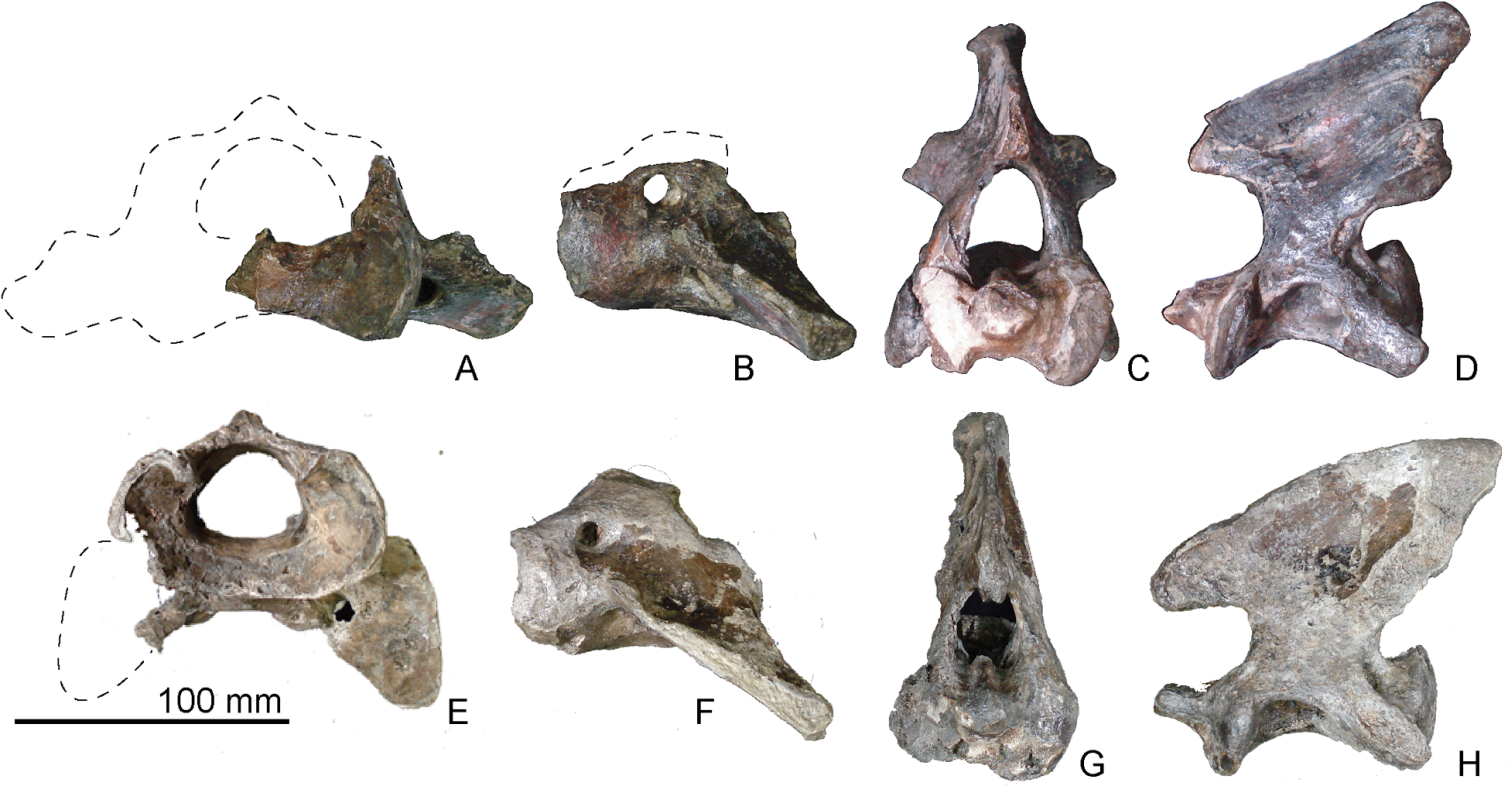
The atlas and axis of *Archaeodobenus akamatsui* and *Pseudotaria muramotoi*. (A)—(D) *Archaeodobenus akamatsui*, (E)—(H) *Pseudotaria muramotoi*, (A) and (E) anterior view of the atlas, (B) and (F) left lateral view of the atlas, (C) and (G) anterior view of the axis, (D) and (H) left lateral view of the axis.

**Fig 10:**
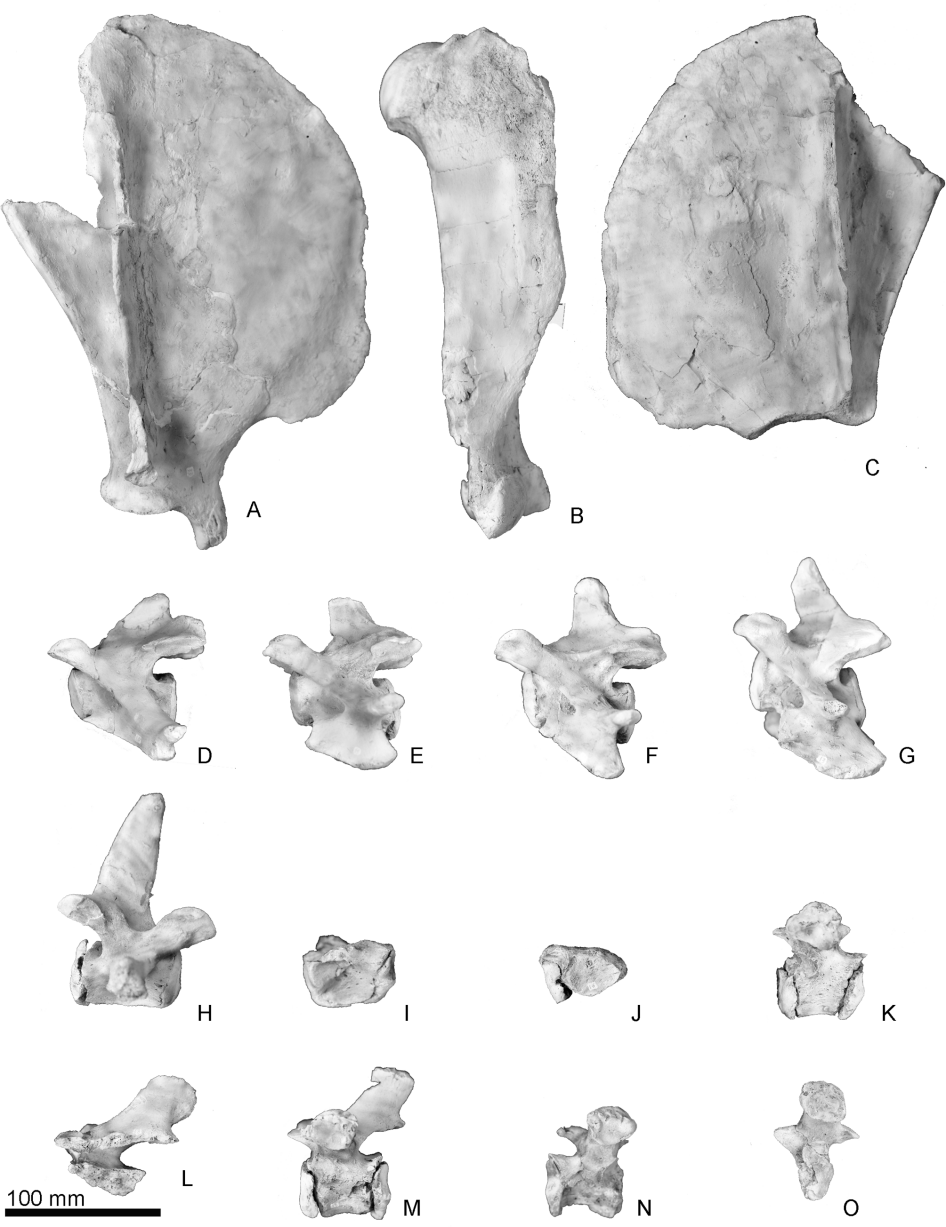
The scapulae, humerus and vertebrae of *Archaeodobenus akamatsui*. (A) lateral view of right scapula, (B) lateral view of right humerus, (C) lateral view of left scapula, (D)—(H) left lateral view of the cervical vertebrae, (D) third, (E) fourth, (F) fifth, (G) sixth, (H) seventh, (I)—(O) left lateral view of the thoracic vertebrae, (I) first, (J) second, (K) third, (L) fourth, (M) fifth, (N) sixth, (O) seventh.

**Table 1:** Measurements (mm) of the crania of *Archaeodobenus akamatsui*, sp. nov. Parentheses indicate measurements that are as preserved. Brackets indicate measurements explained by Sivertsen (1954:18–20).

Condylobasal length [0]	304
Zygomatic width [17]	105.8
Prosthion-palatal notch	—-
Postpalatal length (palatal notch-basion)	—-
Basion to anterior edge of zygomatic root [18]	198.8
Basion to anterior edge of glenoid fossa [21]	98.4
Length of tooth row, C to M1	95
Length of tooth row, P1 to M1	70.6
Greatest width of anterior nares [3]	—-
Greatest hight of anterior nares	—-
Greatest length of nasals [4]	—-
Width of rostrum across canines [12]	78.6*
Width between infraorbital foramina	—-
Width of zygomatic root of maxilla [14]	24.1
Width across antorbital processes [5]	—-
Width across greatest interorbital constriction [6]	—-
Width across supraorbital processes [7]	—-
Width of braincase at anterior edge of glenoid fossa [8]	—-
Width of palate between anterior root of of P1	240
Width of palate between anterior root of of P2	328
Width of palate between anterior root of of P3	314
Width of palate between anterior root of of P4	310
Width of palate between anterior root of of M1	264
Width of palate behind pterygoid process	—-
Transverse diameter of infraorbital foramen	13.2
Vertical diameter of infraorbital foramen	14.6
Auditory width [19]	40.3†
Mastoid width [20]	73.7†
Paroccipital width	103.4†
Greatest width across occipital condyles	71.7
Greatest width of foramen magnum	26.9
Greatest height of sagittal crest	—-
Mandible	
Total length	220
Length of mandibular teeth row (incisive-molars incl.)	196.8
Width between outer sides of condyles	40.4*
Length of cheek teeth row, at crone	63.8
Height at meatus, at just posterior to m2 alveoli	41.9
Heght of coronoid process from angular process	76.1

† indicates estimated measurements.

* indicates estimated transverse measurements that are half-cranium measurements multiplied by two.

**Table 2:** Measurements (mm) of the alveoli of the toothrow of *Archaeodobenus akamatsui*, gen. et sp. nov., holotype.

I1 anteroposterior diameter of alveolus	11.2	13.1
I1 transverse diameter of alveolus	4.8	5.0
I2 anteroposterior diameter of alveolus	12.5†	11.6†
I2 transverse diameter of alveolus	7.4	7.4
I3 anteroposterior diameter of alveolus	18.5	17.0†
I3 transverse diameter of alveolus	11.6	9.8†
C anteroposterior diameter of alveolus	26.5†	—-
C transverse diameter of alveolus	20.6	—-
P1 anteroposterior diameter of alveolus	11.0	—-
P1 transverse diameter of alveolus	9.0	—-
P2 anteroposterior diameter of alveoli	11.5	—-
P2 transverse diameter of anterior alveolus	6.1	—-
P2 transverse diameter of posterior alveolus	9.0	—-
P3 anteroposterior diameter of alveoli	12.5	—-
P3 transverse diameter of anterior alveolus	7.3	—-
P3 transverse diameter of posterior alveolus	9.0	—-
P4 anteroposterior diameter of alveoli	11.2	—-
P4 transverse diameter of anterior alveolus	5.8	—-
P4 transverse diameter of posterior alveolus	8.9	—-
M1 anteroposterior diameter of alveoli	11.6	—-
M1 transverse diameter of anterior alveolus	5.3†	—-
M1 transverse diameter of posterior alveolus	5.3†	—-
M2 anteroposterior diameter of alveoli	9.6	—-
M2 transverse diameter of anterior alveolus	5.4	—-
M2 transverse diameter of posterior alveolus	4.5	—-
Lower		
i2 anteroposterior diameter of alveolus	9.7†	—-
i2 transverse diameter of alveolus	3.9†	—-
i3 anteroposterior diameter of alveolus	11.4†	9.4†
i3 transverse diameter of alveolus	7.6†	5.4†
c anteroposterior diameter of alveolus	28.3	29.1
c transverse diameter of alveolus	18.5	15.2
p1 anteroposterior diameter of alveolus	8.9	8.4†
p1 transverse diameter of alveolus	7.9	6.9†
p2 anteroposterior diameter of alveoli	12.3†	11.8†
p2 transverse diameter of anterior alveolus	6.8	9.0†
p2 transverse diameter of posterior alveolus	7.5	8.0†
p3 anteroposterior diameter of alveoli	13.6†	14.0†
p3 transverse diameter of anterior alveolus	6.8	6.8†
p3 transverse diameter of posterior alveolus	7.1	6.5†
p4 anteroposterior diameter of alveoli	10.5†	—-
p4 transverse diameter of anterior alveolus	5.4	—-
p4 transverse diameter of posterior alveolus	5.8	—-
m1 anteroposterior diameter of alveoli	10.7†	—-
m1 transverse diameter of anterior alveolus	6.0	—-
m1 transverse diameter of posterior alveolus	5.4	—-
m2 anteroposterior diameter of alveolus	7.5	—-
m2 transverse diameter of anterior alveolus	4.9	—-

† indicates estimated measurements.

**Table 3:** Measurements (mm) of preserved teeth of *Archaeodobenus akamatsui*, gen. et sp. nov., holotype.

I1 height/width (crown)/length(crown)	21.9/3.6/6.0
I2 height/width (crown)/length(crown)	25.8*/5.9/10.4
I3 (in situ) height (without root)/width (crown)/length(crown)	24.6/11.8/16.2
C (in situ) height (without root)/width (crown)/length(crown)	41.1/19.3/23.8
P2 (in situ) height (without roots)/ width (crown)/length(crown)	8.9/10.0/8.0
P3 height/width (crown)/length(crown)	19.8/8.0/7.2
P4 height/width (crown)/length(crown)	15.6/7.6/8.5
C (in situ) height (without root)/width (crown)/length(crown)	32.0/24.5/15.9
p1 height/width (crown)/length(crown)	17.2*/6.2/7.0
p3 height/width (crown)/length(crown)	21.5/7.2/10.6

* indicates estimated measurements.

#### Diagnosis

*Archaeodobenus akamatsui* is an archaic odobenid with a slender and small cranium and non-tusked, moderate-sized upper canine. Distinguished from other archaic odobenids (*Prototaria primigena*, *P*. *planicephala*, *Proneotherium repenningi*, *Neotherium mirum*, *Kamtschatarctos sinelnikovae* and *Pseudotaria muramotoi)* by the following derived characters: pentagonal basioccipital (character 30), blade-like spinous process of axis (character 91). Distinguished from later diverging odobenids (*Imagotaria downsi*, *Pontolis magnus*, dusignathines and odobenines) by retention of the following primitive characters: deep mandibular fossa (character 26); small mastoid process (character 33); two distinct roots on premolars (expect P1) (character 74 and 76).

#### Holotype

UHR 33282: Hokkaido University Museum, associated partial skeleton including a partial cranium (mostly left half) with left and right I3, right C, right P2, a left mandible with canine, a partial right mandible with incomplete canine, a basihyoid, a left thyrohyoid, ceratohyoids, nearly complete atlas, axis, cervical vertebrae 3–7, incomplete thoracic vertebrae 1–8, a left rib, three right ribs, an incomplete sternebra, partial scapulae, and nearly complete right humerus.

#### Type Locality

UHR 33282 was collected as a float near the Rutaka Bridge on the Tobetsu River by staff of the Tobetsu Town Board of Education. It is 1.5 km south and also down stream from Aoyama dam in Tobetsu-cho, Ishikari-gun, Hokkaido, Japan (43°27’34” north latitude and 141°35’35” east longitude) (Fig 2).

#### Formation and Age

UHR 33282 was enclosed in a weathered, dark green fine-grained, massive consolidated glauconitic sand stone block as a float, originally from the glauconite bed from the top of the Ichibangawa Formation. Although the Lower Pliocene Tobetsu Formation is exposed at the locality [1], it is very different from the matrix adhering to the new fossil, suggesting that the block was transported from the upstream of the river. Three formations (the Morai, Ichibangawa and Ponsubetsu Formations) are exposed further upstream the Tobetsu River (Fig 2) [1]. But these formations are silt, except the Ichibangawa Formation that is sandstone. In addition, the rich-occurrence of glauconitic sandstone with marine fossils and carbonates is known only in the upper part of the Ichibangawa Formation in this area (Kakimi and Uemura [2]), as shown by the gray band in Fig 2. Thus, the origin of UHR 33282 is the glauconite bed on the top of the Ichibangawa Formation.

Recognition of the glauconite bed is disputed. Kakimi and Uemura [2] described the bed as the uppermost of the Ichibangawa Formation. The Kabato Collaborative Research Group [1] and Takano et al. [3] recognized the bed as the lowermost part of the Morai Formation. On the other hand, Takano et al. [4] recognized as a separate unit and did not include it in both the Ichibangawa and Morai Formations. The lithology of the matrix, fine-grained, massive hard sand stone is similar to the Ichibangawa Formation (sandstone), rather than the Morai Formation (shale siltstone). In short, here, we follow the oldest study; i.e., Kakimi and Uemura [2], to recognize the fossil odobenid’s original formation, and also follow the estimated age of the glauconite bed of Takano et al. [4].

As Fig 2 shows the age of the Ichibangawa Formation is lacking. However, the overlying Morai Formation includes the *Denticulopsis katayamae* diatom zone, 9.2–8.5 Ma [5]. Also, the underlying Subetsu Formation has a fission track age of 13±1 Ma by Tsuji [6], but it is not exposed at the locality [1]. Based on these chronological information, Takano et al. [3] estimated the age of the Ichibangawa Formation (except the glauconite bed) to be approximately 11.5–10 Ma, and they also considered the uppermost glauconite bed of the Ichibangawa Formation to be around 10–9.5 Ma based on previous studies.

#### Etymology

The species is named in honor of Dr. Morio Akamatsu, a curator emeritus of the Hokkaido Museum, for his longstanding contributions to geology and paleontology of Hokkaido, and in gratitude for his encouragement and assistance to both of us throughout this study.

## Description

**Fig 11:**
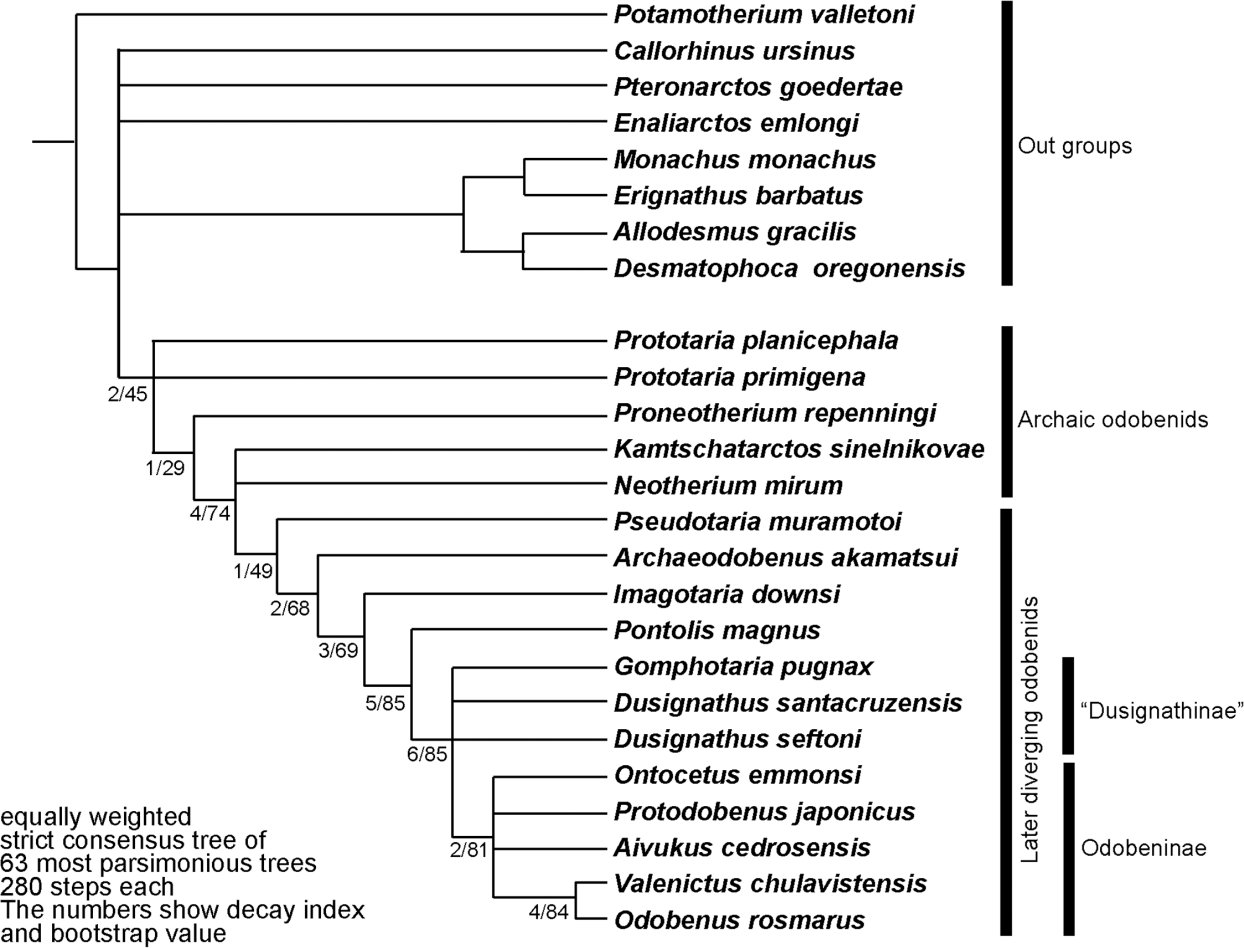
Reconstruction of *Archaeodobenus akamatsui* by Tatsuya Shinmura (Ashoro Museum of Paleontology).

Morphological terms follow Mead and Fordyce [7] for postcrania. Measurements are in Tables 1–3. A life reconstruction is presented in Fig 11.

### Gender and ontogenetic age

The nine cranial sutures that Sivertsen [8] considered to be useful to evaluate relative ontogenetic ages of otariid pinnipeds, at least six are nearly closed. This indicates that the new fossil belongs in Sivertsen’s Group II age class (i.e., subadult). In addition, well developed lambdoidal crests and relatively large canines suggest that the animal represents a male.

#### Estimated body size

The estimated body size of the holotype individual is 2.8–3 m in total length and 390–473 kg in total weight. Holotype of *A*. *akamatsui* is an intermediate size between modern male *Eumetopias jubatus* (3.3m, 1000kg) and *Otaria flavescens* (2.6 m, 300–350 kg) (Jefferson et al. [9]).

The body size of *Archaeodobenus akamatsui* can be inferred by the method of Churchill et al. [10], using formulae for the Otariidae (S1 File). The holotype cranium of *A*. *akamatsui* shows similar proportion with otariids rather than highly specialized modern walrus *Odobenus rosmarus*. On the holotype of *A*. *akamatsui*, the posterior end of the palatine is missing, which is used as a landmark to apply the formulae. Here, we took two measurements for the palatine length from the prosthion to the most posterior preserved point of the palatine and the most anterior preserved point of the hamular process base. Thus, the estimated body length and mass are presented as a range of possible body size.

#### Cranium

(Figs 3–5; Tables 1 and 2)

#### Occipitals

The occipitals are preserved mainly on the left side of the cranium. The basioccipital is broad posteriorly and trapezoidal in shape. The muscular tubercle for the origin of the longus and rectus capitis muscles is well developed anterolaterally on the midportion of the basioccipital. The portion posterior to this tubercle bears a deep and anteroposteriorly long hemispherical fossa. The jugular (posterior lacerate) foramen is large (12.0 mm long and 11.2 width) and crescentic in shape, but this might be a result of deformation, a weak slide to the medial. The hypoglossal foramen is relatively large, and its diameter is 4.6 mm. The occipital condyles are transversely narrow and dorsoventrally high, and are projected weakly posteriorly. The medial margins of the occipital condyles from posterior view are nearly vertical. The foramen magnum is dorsoventrally deep and elliptical in shape. The dorsal and ventral condyloid fossae are deep. A pair of crests (around 20 mm length) rise up from the medial side of the occipital condyles to the middle, on the dorsal margin of the foramen magnum uniquely (Fig 5). The jugular process is short, mediolaterally thin, and plate-like in structure. The suture between the jugular process and mastoid process is not fused. The nuchal tubercle is very weak. The occipito-squamosal suture is almost fused. The lambdoidal crest is thick and posterodorsally projected.

#### Squamosal

The left squamosal is nearly complete, but missing the anterior part of the slender zygomatic process. The pseudosylvian sulcus is weakly present, and it is located just above the temporal fossa. The temporal fossa, between the braincase and the zygomatic process, is relatively wide, smooth and is continued posteriorly to the dorsal surface of the laterally projected mastoid process, so that the temporal fossa has an anteroposteriorly extended flat floor of 38.3 mm in length. The mandibular fossa is subcylindrical, and it makes a deep groove dorsally. The retroglenoid process is a transversely wide and anteroposteriorly thin plate, which projects strongly anteroventrally and forms deep mandibular fossa. The mastoid process is relatively small and projected laterally. Its lateral surface for the origins of the sternomastoideus and cleidomastoideus muscles is smooth probably because of the young age of the holotype individual.

#### Tympanic Bulla

The ventral surface of the tympanic bulla is inflated and smooth. The tympanic bulla is comprised mostly of ectotympanic and delimited anterolaterally by the posteromedial side of the retroglenoid process and posterolaterally by the anteromedial side of the mastoid process. An anterior part of the bulla has a mediolaterally elongated shallow depression, just posterior to the retroglenoid process. The external acoustic meatus is relatively large and circular, approximately 7.8 mm in diameter. The stylomastoid foramen opens ventrally at the point medial to the mastoid process. The hyoid fossa opens at the posterolateral end of the tympanic bulla and it is 7.5 mm long and 5.0 mm wide. Slightly medial to the hyoid fossa, the carotid canal opens as size is 6.7 mm long and 4.9 mm wide. The anterior opening of the carotid canal is circular, moderate (4.5 mm diameter) and located at the anteromedial margin of the tympanic bulla.

#### Petrosal

The middle ear region of a long hexagonal petrosal is visible on the ventral floor of the braincase. Its anterior portion is positioned ventrally and is wider than its posterior portion. The petrosal crest is observed on the anterior part of the petrosal. A deep transverse sulcus lies on dorsal border of the petrosal, just below to the bony tentorium. The cerebellar fossa is dorsoventrally elliptical and has a small slit on the ventral part of the fossa. There is a knob-like tubercle on the medial margin of the cerebellar fossa. The internal acoustic meatus (internal auditory meatus) is anteroposteriorly elliptical, and the entrances for the cranial nerves VII and VIII are separated within the meatus.

#### Sphenoids

The left side of the sphenoids are partially preserved, but the presphenoid and the dorsal half of the alisphenoid are missing. The ventral half of the alisphenoid forms the posterior wall of the choanal trough. The anterior opening of the alisphenoid canal is observed on the medial wall of the alisphenoid at the position of the orbital fissure. The posterior opening of the alisphenoid canal and the foramen ovale are joined in a common recess, and it is located just posterior to the laterally expanded pterygoid strut at the medial side of the mandibular fossa.

#### Pterygoid

The pterygoid strut is dorsoventrally and transversely broad. There is a sharply edged ventromedial ridge present on the ventral surface of the pterygoid that extends from the posterior end of the palate to the long and slender, posteroventrally projected pterygoid hamulus.

#### Maxilla

The medial part of the palatal portion of the maxilla is broken away, but enough of that portion remains to show a transversely arched palate. The palatal margin of the maxilla is laterally divergent at the portion of M1. The infraorbital foramen is relatively small and dorsoventrally elliptical. The infraorbital canal runs through rather thick bone at the maxillary foramen. The dorsal roof of the infraorbital foramen is dorsoventrally thick. The orbit is large, however the medial wall of the orbit is broken away. On the ventral surface of the zygomatic root, there is a shallow fossa and a weakly developed ventral tuberosity.

#### Premaxilla

The dorsal part of the premaxilla is not preserved. The rostral process is large and blunt. The palatine fissures (premaxilla foramina) are separated by a thin septum at the mid-portion, and each foramen is anteroposteriorly elongated (14.4 mm long and 5.6 wide on the right side).

#### Palatine

The lateral half of the left palatine is preserved. The palatine is anteroposteriorly long, but its posterior borders with the pterygoid and the sphenoid are not clear.

#### Jugal

The zygomatic arch of the jugal is long, thicker anteriorly and thinner posteriorly. Laterally, the zygomatic arch has a weakly curved and rounded orbital margin, a well-developed frontal process (postorbital process) on the dorsal margin, and a weakly curved masseteric margin ventrally. The outline of the anteroventral process is not clear because the maxilla-zygoma suture is closed. Posterior end of the temporal process projects dorsoventrally and rounded.

#### Mandible

The mandibular symphysis is opened, rugose and is dorsoventrally long and elliptical. The posterior end of the mandibular symphysis is same level at the p2 alveoli. In dorsal and ventral view, the body of the mandible is slightly skewed laterally on its medial portion, just posterior to the m2 alveolus. The diastema between the canine and the first premolar is short. In lateral view, the dorsal and ventral borders of the mandibles are nearly parallel, but they diverge posterior to the coronoid process of the ascending ramus. The rami of the mandibles are V-shaped in dorsal view and curved posteriorly at m1. The anterior mental foramen is rounded, small (3.4 mm diameter) and located just beside the mandibular symphysis. The middle mental foramen is also circular, large (4.4 mm diameter) and located beneath p1. The posterior mental foramen is smaller (3.1 mm diameter) than both the anterior and middle mental foramina and located beneath p2. At the level of p2 and p3, a well-developed genial tuberosity is present on the ventral margin of the body. A narrow coronoid process is thinner dorsally, rounded and anterodorsally projected. A wide (39.2+ mm) and smooth condyloid process is present at the level of the tooth row. The mandibular notch opens wide anterodorsally. The angular process is weakly projected posteriorly and forms a small medial shelf. The masseteric fossa is shallow and its borders are unclear, with a weak horizontal crest at the dorsal part of the fossa. Medially, the mandibular foramen is small, dorsoventrally elliptical and directed anterodorsally. The mandibular canal, which is at the center of the broken surface of the right mandible, is also elliptical.

#### Dentition

The dentition is represented by upper and lower alveoli on the left maxilla and both dentaries as I1-3, C, P1-4, M1-2, i2-3, c, p1-4 and m1-2. In addition, both I3s, right C and P2, and the lower canines are preserved in place. Isolated left I2 and P3, and the right P4 are also preserved.

The alveoli for I1 and I2 are transversely narrow but anteroposteriorly wide. The crown of the I2 consists of an anteriorly located large cusp and posteriorly located small cusplet. The I2 root is laterally thin and anteroposteriorly wide, and it is gently curved posteriorly. There is a distinct constriction between the crown and the root. A much larger isolated left I2 has a shallow groove on the lateral and proximal surface and is weakly curved posteriorly.

The strongly and anteriorly curved I3s have worn ends and are oval shape. The anterior surface of the crown is smooth, but the posterior surface makes a vertical crest.

The canine is not tusk-like, but large and conical, and it has a strong posterior crista. The root has a broken surface of the maxilla, which is very long and large (86.3 mm long in total of the canine), and transversely narrow. The proximal end of the root is visible from the broken cranium (see Fig 3A), and has an open pulp cavity.

The premolars are double rooted except for the P1. The P1 has a relatively large single root. The P2 has a narrow anterior and a wide posterior alveolus, and its crown has one small main cusp with a blunt lingual cingulum with several incipient cusplets. The P2 to M2 have narrow subcircular anterior alveoli and wide oval posterior alveoli.

Isolated left P3 and right P4, crowns have a posteromedially placed protocone shelf. They are similar in shape, but the P3 is slightly higher in crown height. The P3 has a posterolingual cingulum with one distinct cusp (protocone shelf) at the posteromedial margin of the cingulum. The P3 has a small cusp at the posterior end of the crown. The P4 has a more expanded protocone shelf and relatively large and high cusp also at the posterior end of the crown. It is anteroposteriorly slightly enlarged, forming a small blade-like ridge that is separated from the main cusp by a distinct notch.

The M1 is double rooted, and the posterior alveolus lies outside the general alignment of the cheek teeth. The alveoli of i2 and i3 are mediolaterally thin, anteroposteriorly long, and they are single rooted. The i2 alveolus is smaller than i3.

The lower canine is nearly equal size, but slightly shorter dorsoventrally and narrower transversely than the upper canine, and has a well developed posterior crista. Its root is almost oval with a weak longitudinal groove on the buccal side in cross section and is oriented dorsally.

The p2—m1 are double rooted, while p1 and m2 are single rooted. The p1 to p3 have bulbous crowns with well-developed enamel. An isolated left p3 is preserved, and possesses three distinct cusps; an anteriorly located developed cusp (paraconid), a main cusp (protoconid), and a small but distinct posterior cusp just behind the main cusp. The anterior cusp is smaller than the posterior cusp. The lingual cingulum is well developed and its surface is slightly crenulated. Its root is wider than the crown. The tooth row is short (63.6 mm).

#### Hyoid

The basihyoid, the left thyrohyoid and the ceratohyoids are preserved (Fig 8). The basihyoid is thickened toward both the proximal and distal extremities It has two small joints on the posterior margin for the thyrohyoid The thyrohyoid is missing part of the distal extremity, but its proximal extremity is thickened. The ceratohyoid is slender and it is thickened toward both the proximal and distal extremities. The proximal extremity of the ceratohyoid is about twice as wide as the distal extremity, and it has a flat edge and a nearly perpendicular articulation.

### Vertebral column

#### Atlas

The wing process is large and extends ventrolaterally. The ventral tubercle of the ventral arch is weakly inflated ventrally. The lateral vertebral foramen is small and circular, and it is visible only in lateral view. The transverse foramen is also small; it is anteriorly circular and posteriorly triangular. The atlantal fossae are relatively weak, forming depressions ventral to the base of the wing. Each intraosseous canal opens posteriorly into the anterior transverse foramen. The transverse foramen is relatively large, and the odontoid fovea is also large and deep. Two shallow grooves extend transversely on the posterior surface of the ventral arch. A small and deep foramen is present on the mid-line of the posterior articular surface.

#### Axis

The axis is nearly complete, missing parts of the right anterior articular surface and anterior spinous process. The dens is large and thick. The blade-like spinous process extends posterodorsally. The dorsal margin of the spinous process is straight and anteroposteriorly long. The posterior edge of the spinous process expands laterally. The vertebral foramen is large and elliptical. The transverse process is large, and it is projected posterolaterally and ventrally. The transverse foramen is large and circular, and they are located at the base of the transverse process. On the ventral surface of the vertebral body, a pair of deep fossae are divided by a median crest. The anterior end of the median crest is developed as an anteriorly angled triangular tubercle.

#### Third to seventh cervical vertebrae

The third to seventh cervical vertebrae are preserved (Fig 10D–10H). The transverse processes are long and knob-like in the third cervical, transversely thin and plate-like in the forth to sixth cervicals, and very weak knob-like in the seventh cervical vertebra. The spinous processes are projected weakly in the third to sixth cervical and strongly ventrally in the seventh cervical vertebra.

#### Thoracic vertebrae

Seven thoracic vertebrae are preserved although their exact positions are not clear (Fig 10I–10O). The transverse processes are strongly projected in the “third, fifth to seventh” thoracics; squire shaped costal fovea are present on the transverse process. The spinous processes are projected posteroventrally in the “fourth and fifth” thoracics, but other thoracics are not well preserved.

#### Rib

Seven ribs are preserved. The left first to third ribs are nearly complete, but other ribs are preserved only proximal parts. The first rib is the shortest. The head and tubercle are distinct and well separated in each rib. Each rib is gently curved. The body of each rib is somewhat square in cross section, and therefore the anterior and posterior surfaces are relatively flat. The costochondral junction is semicircular and elliptical in cross section.

#### Sternum

Two intermediary sternebrae are preserved. The sternebrae are short and thick, forming a square pole. Lateral thickness is wider than dorsoventral. It is thickened toward the anterior and posterior extremity. Both extremities are display a rugose articulation.

### Appendicular skeleton

#### Scapula

Both scapulae are well preserved (Fig 10A and 10C); the right scapula is nearly complete, missing only a part of the posterior angle, lateral margin of the spine, and the acromion. The dorsal part of the spine is thick and high compared to its ventral part. The base of the acromion extends ventral to the scapular notch. The scapular spine is positioned posteriorly; so that the supraspinous fossa is wider than the infraspinous fossa. Within the supraspinous fossa are two weak ridges parallel to the spine; the anterior one is weakly curved anteriorly, and posterior one is nearly straight. The scapular notch is deep. The anterior border is gradually curved. The posterior border is nearly straight. The scapular tuberosity is large. On the medial surface of the scapula, four muscule attachment ridges are present. Two most posterior lines are merged ventrally, forming a Y-shape.

#### Humerus

The right humerus (Fig 10B) is nearly complete, but missing part of the ventral margin of the lateral epicondyle. The head is transversely small and anteroposteriorly long and elliptical. The greatest length of the head is around 20% of the total length of the humerus. An epiphyseal suture is still persistent at the anterior border of the head. The neck of the humeral head is indistinct. The shaft is relatively straight and slender. The greater tubercle is robust and elevated slightly above the head, and its dorsal surface is rugose. An elliptically shaped surface is present at the apex of the greater tubercle. The lesser tubercle is medially projected and located distinctly below the head. The intertubercular groove is narrow and deep between the greater and lesser tubercles. The tricipital line is present. The deltoid tuberosity is well developed on the pectoral crest. The pectoral crest is straight and directed distally toward the medial lip of the trochlea. The brachial groove is deep. The lateral epicondyle is conspicuously reduced and scarcely projected from the lateral margin of the distal radial capitulum. There is a deep groove on the lateral surface of the lateral epicondyle. The supinator ridge is weak and blunt. The medial epicondyle is knob-like and quadrangular in outline. The epicondylar foramen is absent. The capitulum of the humerus is low proximodistally. The trochlea is mediolaterally short. Both the olecranon and radial fossae are shallow. The supratrochlear foramen is absent. Although the distal tip of the trochlea and the posterodistal part of the capitulum are somewhat broken, the former is much greater in anteroposterior diameter than the latter as inferred from its width.

## Phylogenetic Analysis

### Relationships of the new fossil to other odobenids

The new fossil belongs to the Family Odobenidae in having a laterally expanded pterygoid strut (Character 13), a bony tentorium that is closely appressed to the petrosal (Character 29) and well-developed P1 and P2 with lingual cingula (Character 71). Those characters are recognized as unequivocal synapomorphies for the family [11–13]. Thus, the phylogenetic analysis for the new fossil was focused on members of the Odobenidae with member of other pinniped clades as outgroups. Previously Deméré [14] and Kohno [11, 12] presented cladistic analyses for the family Odobenidae mainly on the basis of cranial, mandibular and dental morphologies. Recently, Boessenecker and Churchill [13] analyzed additional characters of both crania and mandibles with cheek teeth of the odobenids. Although most of these characters were same characters used by Deméré [14], Kohno [11, 12], Kohno et al. [15], and Deméré and Berta [16], they defined all the characters used in their analyses more clearly and thoroughly illustrated on Morphobank (http://morphobank.org/index.php/Projects/ProjectOverview/project_id/530). Accordingly our data matrix is the one by Boessenecker and Churchill [13] with minor changes and an additional axial character (Character 91) for our analysis (S2–S5 Files). We included 17 odobenids and eight outgroup taxa that were used by Deméré [14], Kohno [11, 12], and Boessenecker and Churchill [3], and we also added extinct and early diverging arctoid, *Potamotherium valletoni* Geoffroy, 1833 (following Rybczynski et al. [17]) as an outgroup in this analysis. Among the taxa within Odobenidae, *Pelagiarctos thomasi* Barnes, 1988 and an indeterminate species belonging in the same genus reported by Boessenecker and Churchill [13] from the middle Miocene of California were excluded from our analysis because of the lack of any cranial material for this taxon at present. In total, our analysis used 17 ingroup and nine outgroup taxa coded for 91 morphological and morphometric characters (S2–S5 Files). Both character and tree data were manipulated using Mesquite 2.75 [18]. A cladistic analysis was performed with TNT, 1.1 [19]. All characters were unweighted and unordered. A heuristic search of 10,000 replicates was conducted, using tree bisection reconnection (TBR) and saving 10 trees per replication. To measure node stability, we calculated a bootstrap value using symmetric resampling with 2000 replicates and the decay index [20] for the strict consensus tree.

**Fig 12:**
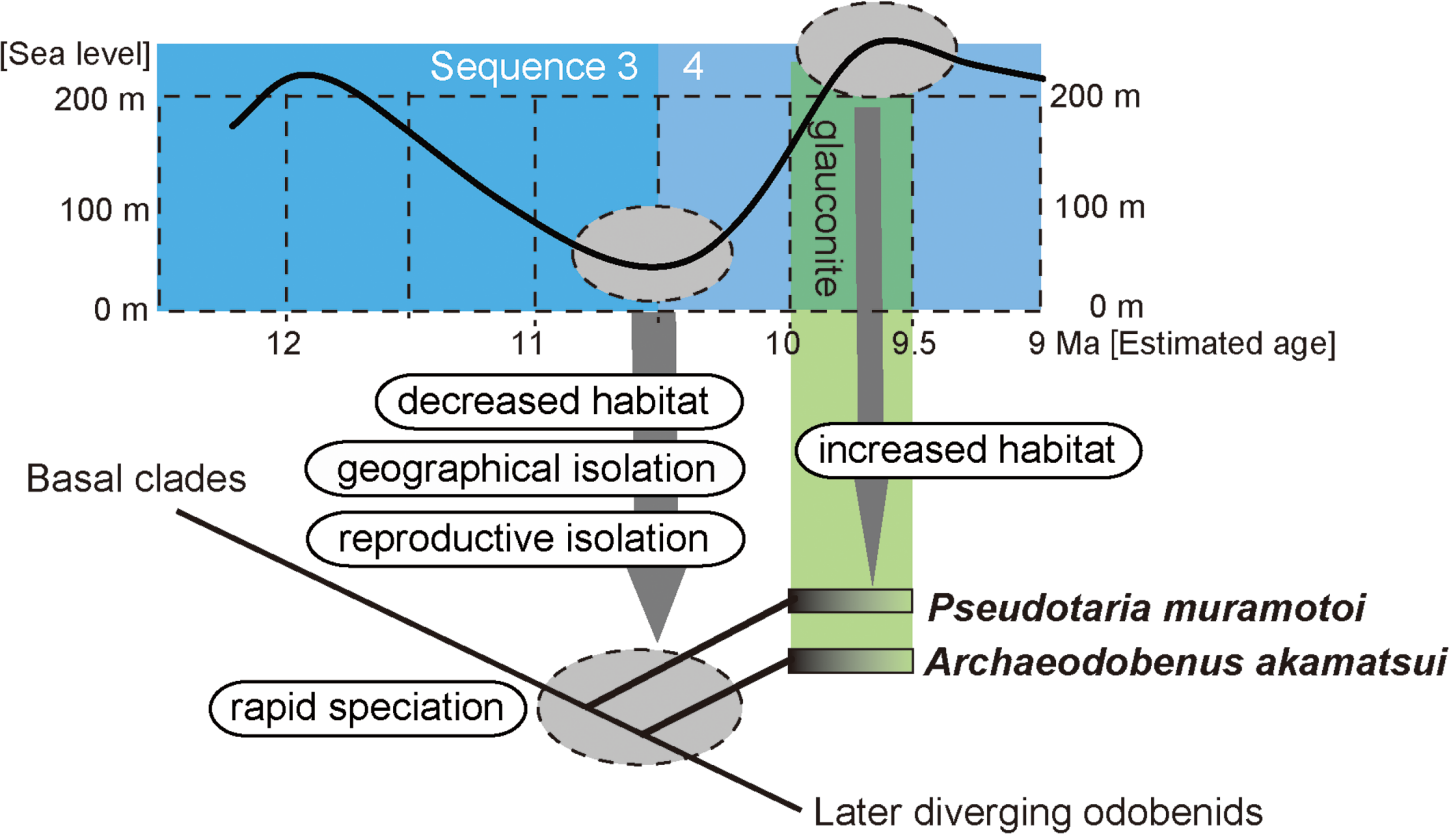
The strict consensus tree of equally weighted analysis of *Archaeodobenus akamatsui* and the Odobenidae, with Bremer support at nodes.

Our analysis resulted in 63 most parsimonious trees with 280 steps. The variations among the 63 trees are represented by differences in branching patterns among outgroup taxa and also ingroup taxa (mostly “dusignathines”) within the later diverging odobenids (see a strict consensus tree in Fig 12). Phylogenetic analysis revealed that the new fossil is recognized as an independent linage. The new fossil is distinguished from such earlier diverging odobenids as *Prototaria* spp, *Proneotherium repenningi*, *Kamtschatarctos sinelnikovae*, *Neotherium mirum* and *Pseudotaria muramotoi* in having two unequivocal and equivocal synapomorphies with later diverging odobenids such as the pentagonal basioccipital (Character 30) and straight dorsal margin of the spinous process of the axis (Character 91) respectively. However, the new fossil lacks synapomorphies of later diverging odobenids (i.e., *Imagotaria downsi*, *Pontolis magnus*, the “dusignathines” and the Odobeninae) such as the presence of ventral tuberosity of the zygomatic root of the maxilla (Character 7), shallow glenoid fossa (Character 26), large mastoid process of the squamosal (Character 33), absence of the metaconid on the lower cheek teeth (Character 70) and double rooted upper P3 roots (Character 74). Although not included within the computer-based phylogenetic analyses, *Pelagiarctos thomasi* and *P*. sp. of Boessenecker and Churchill [13] are clearly distinguishable from the new fossil by their very large size and robustness and by having extremely large cheek teeth. Therefore, the new fossil is differentiated from all previously known taxa within the Odobenidae. In addition, the new fossil has autapomorphic characters such as sharply divergent mandibular arch (Character 44) and slightly concave talonid basin of the lower cheek teeth (Character 69).

These distinctive features of the new fossil among taxa within the Odobenidae indicate that this taxon is monophyletic (Fig 12). Therefore, the generic level assignment as a new taxon is warranted for the new fossil described here. Thus, we propose *Archaeodobenus akamatsui* as a new genus and new species within the family Odobenidae.

## Discussion

### Disparity of Archaeodobenus akamatsui and Pseudotaria muramotoi

*Archaeodobenus akamatsui* gen. et *sp*. nov. is presently known only from the holotype, which is from the top of the Ichibangawa Formation as described above. The same formation has also produced another odobenid *Pseudotaria muramotoi* Kohno, 2006. Therefore, both species are sympatric at least geochronologically. These two species of fossil odobenids are closely related to each other based on the phylogenetic analysis, but are not monophyletic. Indeed, the basioccipital in *A*. *akamatsui* is pentagonal in shape as a derived condition of all the later diverging odobenids, but the same portion in *P*. *muramotoi* is primitively parallel sided as in earlier diverging odobenids as well as the late Oligocene and early Miocene pinnipedimorphs. In addition, the mastoid process is small in *A*. *akamatsui* among the Odobenidae, but is stronger in comparison with the sister *P*. *muramotoi*. Also, the preserved cervicals in this new taxon show morphological differences between them (Fig 9). For example, the blade-like dorsal margin of the spinous process of the axis (Character 91) in *A*. *akamatsui* is recognized as a derived condition that unites this taxon with later diverging odobenids such as the modern walrus, *Odobenus rosmarus* and the Pliocene extinct walrus, *Valenictus chulavistensis* (SDSNH 36786: San Diego Natural History Museum) of the subfamily Odobeninae as the potential synapomorphy for all the later diverging odobenids such as *Imagotaria*, *Pontolis* and “dusignathines” as well as the monophyletic odobenines.

Our analysis also indicates that *P*. *muramotoi* does not have its own character (autapomorphy) and therefore the least modified condition in the transformation series of characters to the later diversing odobenids as suggested also by Kohno [12]. In this sense, these differences between the two taxa within the same family in the same geologic age and same geographic range suggest that *A*. *akamatsui* might have split from *P*. *muramotoi* as a different species, with the rapid structural divergences during a short time period in the late Miocene in the western North Pacific.

### Isolation and speciation of Archaeodobenus akamatsui and Pseudotaria muramotoi

The two different genera and species of the above mentioned fossil walruses from the same formation in a restricted geographical area suggests rapid diversification of later diverging odobenids, among the early late Miocene (between 10.0 and 9.5 Ma) around the Western North Pacific realm. In such case, knowledge of the depositional sequences of the sedimentary basin may be important to recognize cause of rapid diversification in marine mammals (e.g., Pyenson et al. [21]).

The depositional sequences at the locality area was studied by Takano et al, [4]. According to them, the sea-level during the early late Miocene of the locality area had changed dramatically. In the beginning of the early late Miocene (12.5–10.5 Ma), the sea-level was receding, and the shelf structures at the locality had developed (Sequence 3, high stand phase). In addition, development of the fan delta system is known at this sequence. The sea-level during Sequence 4 (from 10.5 Ma) was receding then sudden transgression was initiated around 10.0–9.5 Ma. This sea-level rise is simultaneous with the condensed glauconite layer, from which both *A*. *akamatsui* and *P*. *muramotoi* were found. An association between glauconite and sea-level transgression has been previously reported [22, 23].

**Fig 13:**
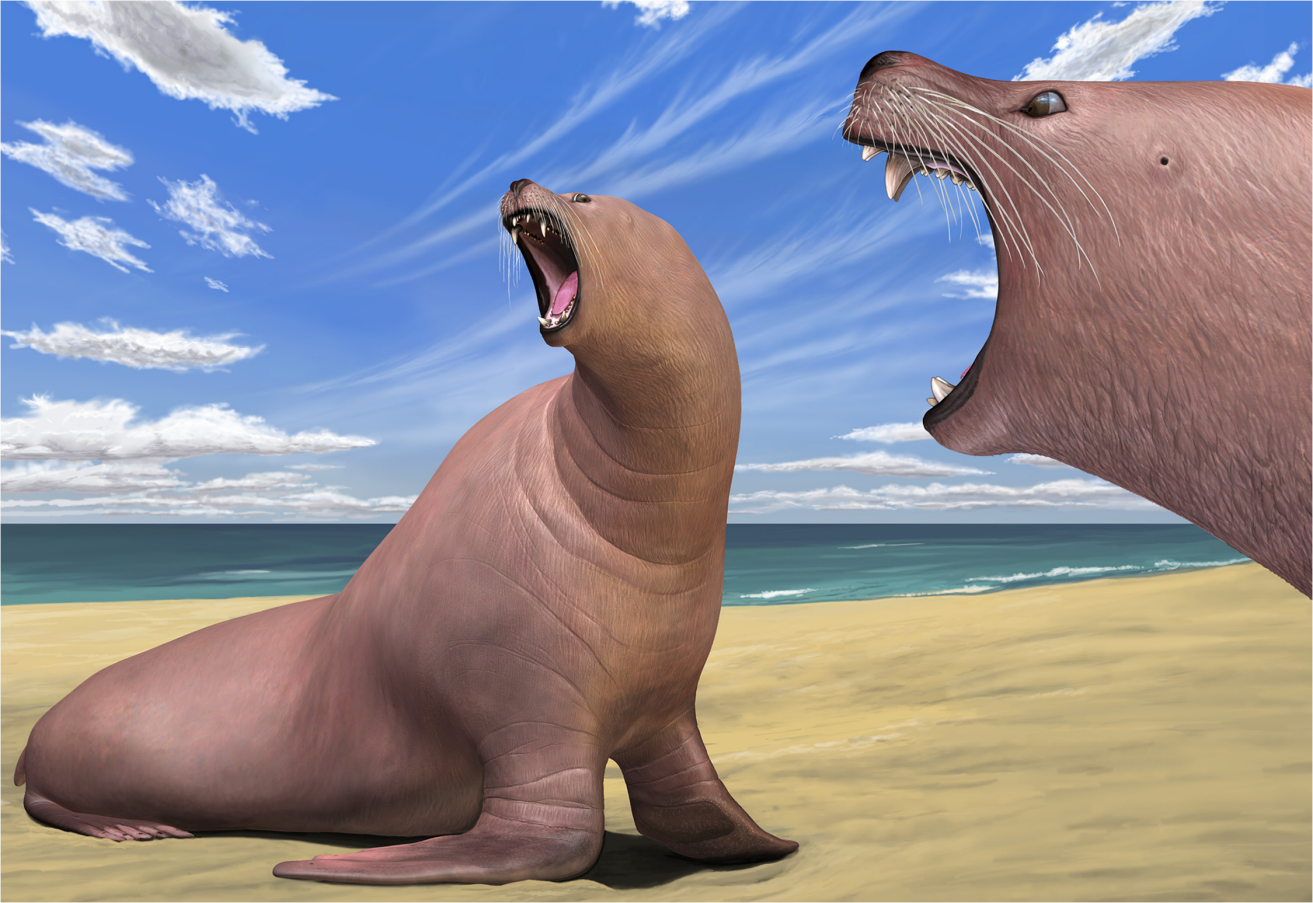
The role of eustasy in early late Miocene odobenid diversification in Hokkaido, Japan.

Considering the relationships between the sea-level changes and the diversification of the archaic odobenids, the occurrence of a regression phase just before the transgression may have been a driving force for the odobenid speciation during the late Miocene (see Fig 13). Firstly, during the transgression of Sequence 3, a common ancestor of both *Pseudotaria muramotoi* and *Archaeodobenus akamatsui* might have occupied a wide range along the western North Pacific coast, tied to availability of shoreline habitat for reproduction. Then, from the end of Sequence 3 to the beginning of Sequence 4 (11.0–10.0 Ma), the sudden sea-level fall resulted in reproductive isolation driven by considerable loss of their shelf habitats.

In contrast, Valenzuela-Toro et al [24] proposed that large-scale marine transgression was linked to local phocid extinction in the late Pliocene of Chile. Changing size of pinniped habitats and degree of sea-level can be considered with the chronological changes of the topography in different areas. The *A*. *akamatsui* locality was situated in shallow marine and close to the margin of the basin sloop [3]. The land area decreased during the transgression stage, which could provide shallow shelf and suitable feeding habitat for the fossil odobenids. The area studied by Valenzuela-Toro et al [25] had large scale transgression, which resulted in much deep water, which were not suitable for the habitat of the pinnipeds.

There may be other explanation for odobenid rapid speciation and diversification during the early late Miocene. Based on the last record of contemporaneous desmatophocid pinnipeds from the early late Miocene Montesano Formation [25], these enigmatic pinnipeds might have reduced in population size and became extinct in the North Pacific [26] in the same period of the possible divergence time of *Pseudotaria* and *Archaeodobenus*. The estimation of the rapid speciation and diversification of these odobenids during the same time period might have been the evidence of the exchange of niche between the two families in the North Pacific. This may also be plausible but is not corroborated by sufficient evidence at present.

Also, Suto et al. [27] has discussed correlations between seqcuences of ocean productivities and evolution of marine organisms during the Neogene and Pleistocene based on the abundances of *Chaetoceros* resting spores (diatoms). According to their results, one of the rich productivities was recognized as the Pacific *Chaetoceros* Explosion Event (PA*C*E-1) in the Late Miocene (i.e., Messinian, ca. 8.5–6.4 Ma), and its incipient increase was also recognized around 10 Ma in the North Pacific. In this regard, Churchill et al. [28] pointed out the productivity and climate change during the Messinian to have strong influence on otariid diversification and range. Although the synchronicity between the increase/decrease of ocean productivities and the marine transgression/regression cycles have not been clear, this may also have related to the fragmentation of the ancestral population and concecutive speciation of *A*. *akamatsui* and *P*. *muramotoi*, and the subsequent increase of the ocean productivity that promoted increase of both populations of two walruses in the locality area.

Newly speciated odobenids diverged allopatrically and migrated or invaded into the”locality area” around 10.0–9.5 Ma during the transgression period. Expansion of their habitats during the transgression just after the regression period allowed the cross over of their existences in the restricted area.

The geographical fragmentation of odobenid population during the period just before the sedimentation of glauconitic layer might have resulted in speciation of the *Archaeodobenus*-like odobenids from common ancestors with the *Pseudotaria*-like odobenids and subsequent increase in size of each population, which may resulted in the diversification of two separate species in the same locality area during the period between 10.0 and 9.5 Ma.

*Pseudotaria* and *Archaeodobenus* among the odobenids can, therefore, be recognized as the beginning of diversification among middle/late Miocene later diverging odobenids that are the two subfamilies Odobeninae+Dusignathinae+*Imagotaria*+*Pontolis*. As discussed above, the driving force of their speciation can be thought as is controlled by the rapid regression, which pushed both geographical and reproductive isolation. The two fossil odobenids from the same formation might show such case of the causal relationship between the sea-level fall and geographical isolation or segmentation.

### Enigmatic odobenid *Pelagiarctos*

We did not include *Pelagiarctos thomasi* from the middle Miocene of California in our phylogenetic analysis because of the lack of its cranial material, but this enigmatic species might have been a member of the “later diverging odobenids” as has suggested by Boessenecker and Churchill [13]. According to them, *Pelagiarctos* shares such derived characters as lateral lower incisors greater in size than medial ones (Character 54) and bulbous postcanine crowns (Character 63) with later diverging odobenids. However, these tooth characters are recognized not only in the odobenids but also in other pinnipeds such as allodesmine desmatophocids [29–31]. On the contrary, *Pelagiarctos* has distinctively double rooted cheek teeth like earlier diverging pinnipeds in spite of having aforementioned derived characters. In this regard, those derived characters may be altered as homoplastic when the cranial material for all the species can be included in the phylogenetic analysis. Therefore, we prefer to reserve judgment temporarily on the phylogenetic position of this enigmatic taxon based only upon the cheek tooth morphologies until the taxon is better understood by cranial material.

## Conclusions

*Archaeodobenus akamatsui* gen. et sp. nov. represents the second taxon of the archaic odobenids from the early late Miocene (ca. 10.0–9.5 Ma) Ichibangawa Formation, Hokkaido, northern Japan. The same formation has also produced the archaic odobenid *Pseudotaria muramotoi* Kohno, 2006. These two odobenids are distinguishable from another in size and shape of the occipital condyle, foramen magnum and mastoid process of the cranium, and some postcranial features. Phylogenetic analysis reveals that *A*. *akamatsui* has two synapomorphies that unites the new species with later diverging odobenids (i.e., *Imagotaria downsi*, *Pontolis magnus*, the “dusignathines” and the Odobeninae). Therefore, two closely similar odobenids having relatively ancestral characters (*P*. *muramotoi*) and relatively derived characters (*A*. *akamatsui*) both lived in the same area during the same age. Perhaps the coexistence of two morphologically similar archaic odobenids as represented by *A*. *akamatsui* and *P*. *muramotoi* in the early late Miocene suggest their rapid speciations by geographical and reproductive isolation during a regressive phase and subsequent transgressions.

## Supporting Information

S1 Appendix (DOCX)

S2 Appendix (DOC)

S3 Appendix (ZIP)

S4 Appendix (ZIP)

S1 File Data of the body estimation of *Archaeodobenus akamatsui*. (DOCX)

S2 File Specimen list. (DOCX)

S3 File Character list. (DOC)

S4 File Data matrix in nexus format. (ZIP)

S5 File Data matrix in TNT format. (ZIP)
