# Correction: A New Late Miocene Odobenid (Mammalia: Carnivora) from Hokkaido, Japan Suggests Rapid Diversification of Basal Miocene Odobenids

**DOI:** 10.1371/journal.pone.0141406

**Published:** 2015-10-20

**Authors:** Yoshihiro Tanaka, Naoki Kohno

The captions for Figs 11, 12 and 13 are incorrectly switched. Please view Figs 11, 12 and 13 and their corrected captions here.

**Fig 11:**
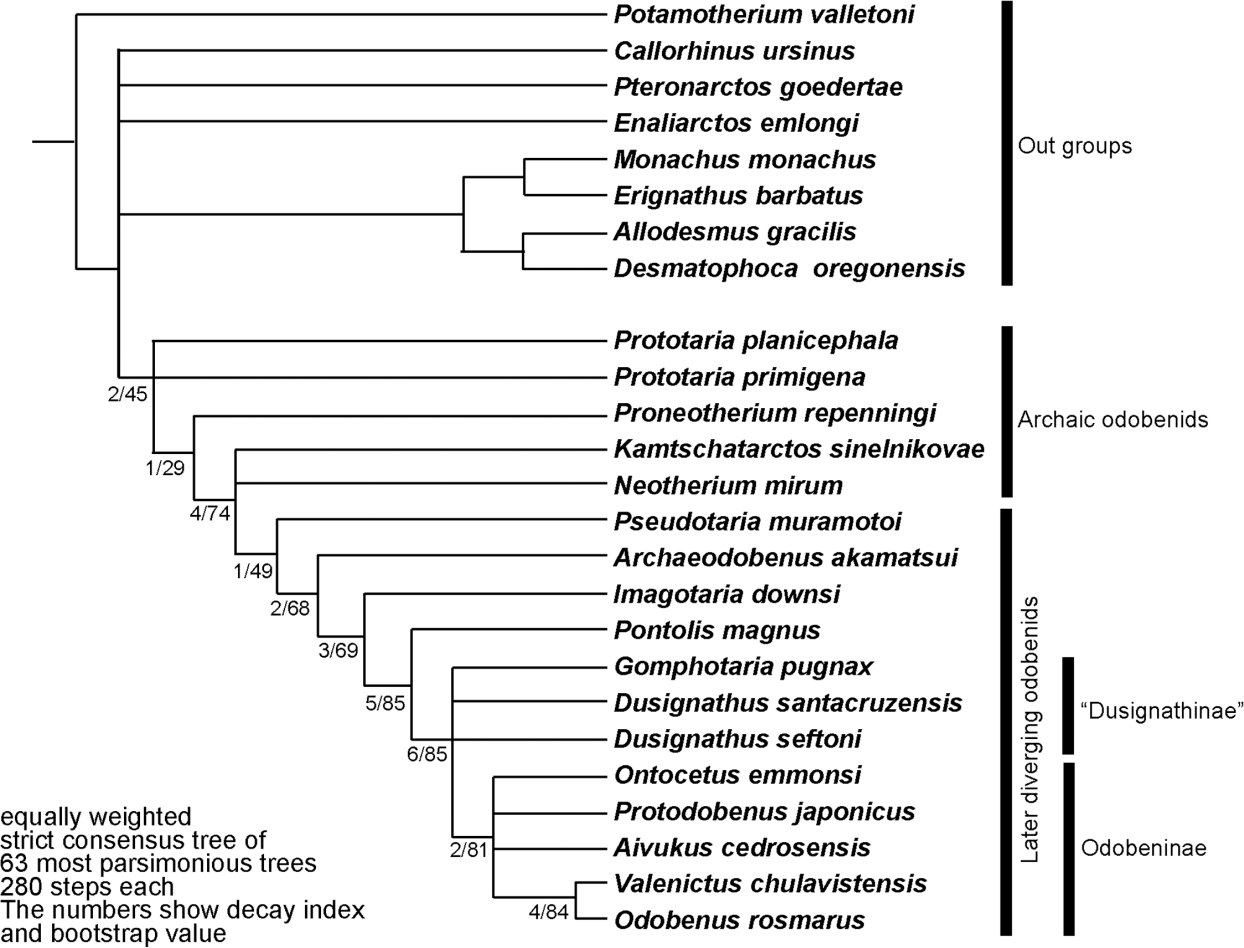
The strict consensus tree of equally weighted analysis of *Archaeodobenus akamatsui* and the Odobenidae, with Bremer support at nodes.

**Fig 12:**
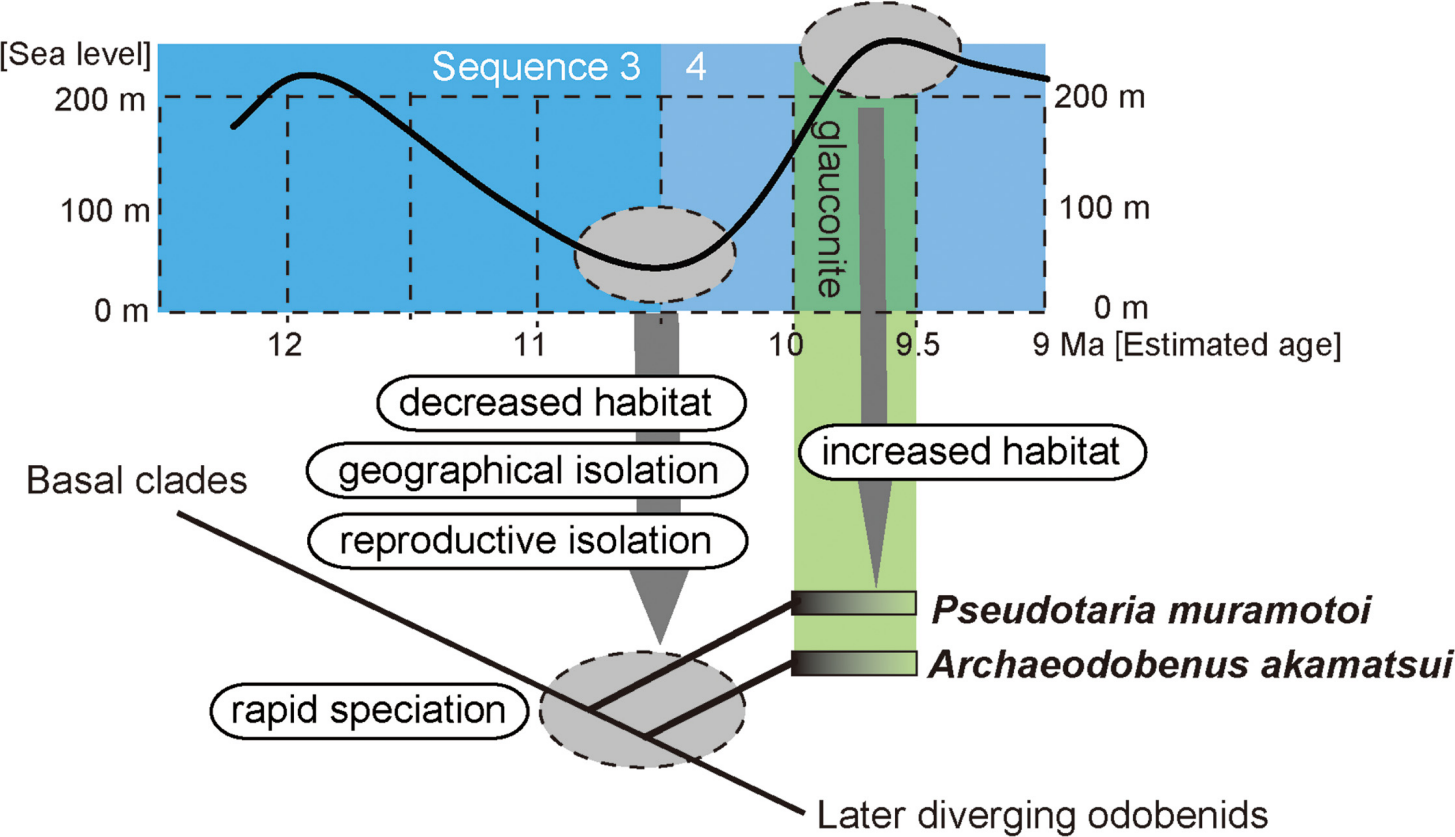
The role of eustasy in early late Miocene odobenid diversification in Hokkaido, Japan.

**Fig 13:**
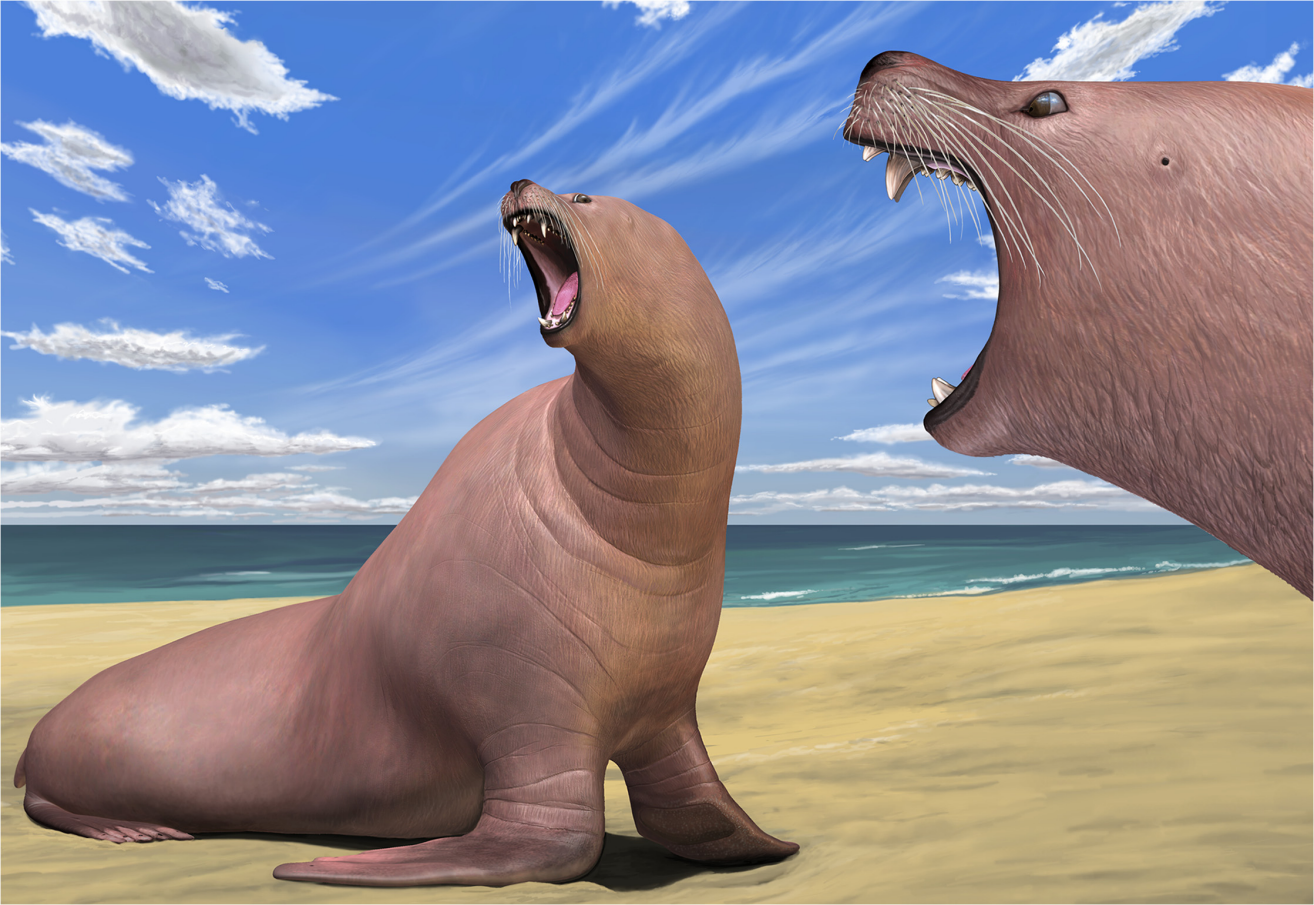
Restoration of *Archaeodobenus akamatsui* by Tatsuya Shinmura (Ashoro Museum of Paleontology)
