# Supplementary material for: A New Late Miocene Odobenid (Mammalia: Carnivora) from Hokkaido, Japan Suggests Rapid Diversification of Basal Miocene Odobenids
Source: PLoS One. 2015 Aug 5;10(8):e0131856. doi: 10.1371/journal.pone.0131856 (PMC4526471; doi:10.1371/journal.pone.0131856)
Supplement: S1 File — (DOCX) [file pone.0131856.s005.docx]

S1 Appendix - The body size of *Archaeodobenus akamatsui* can be inferred by the method of Churchill et al. [[1](#_ENREF_1)].

The formula for body length (all subsets) is 1.9*Log(basal length as 30.4)-0.66*Log(palatal length as between 13 and 15)+0.4. The formula for body mass (step wise) is 10.11*Log(basal length) -1.35*Log(palatal length)-3.23*Log(length of upper tooth row as 9.5)-1.06*Log(width of bulla as 2.6)+3.49*Log (length of orbit as 4.8)-3.60*Log(length of mandible as 22.0)+3.98*Log(length of lower tooth row from canine to m1 as 9.3)-1.90*Log(length of lower post canine tooth row as 6.3)-6.94.

1. Churchill M, Clementz MT and Kohno N (2014) Predictive equations for the estimation of body size in seals and sea lions (Carnivora: Pinnipedia). J Anat 225: 232-245.
