# Supplementary material for: A New Late Miocene Odobenid (Mammalia: Carnivora) from Hokkaido, Japan Suggests Rapid Diversification of Basal Miocene Odobenids
Source: PLoS One. 2015 Aug 5;10(8):e0131856. doi: 10.1371/journal.pone.0131856 (PMC4526471; doi:10.1371/journal.pone.0131856)
Supplement: S2 File — (DOCX) [file pone.0131856.s006.docx]

S2 Appendix - For additional specimens examined, please see Supporting information S1 of Boessenecker and Churchill (2013).

*Archaeodobenus akamatsui*

UHR 33282

*Odobenus rosmarus*

SMAC 2749

NSMTM 36186

*Potamotherium valletoni*

NMNS-PV 20762, 20763, 20830, 20831, 20832, 20833, 20835

USNM 214983

*Prototaria planicephala*

NMNS-PV 20111 (cast of the type specimen SSME 13317)

*Pseudotaria* *muramotoi*

CBM-PV 382 (Type)

NMNS-PV 20135 (Paratype)

*Valenictus chulavistensis*

SDSNH 36786 (Type)
