# Supplementary material for: A New Late Miocene Odobenid (Mammalia: Carnivora) from Hokkaido, Japan Suggests Rapid Diversification of Basal Miocene Odobenids
Source: PLoS One. 2015 Aug 5;10(8):e0131856. doi: 10.1371/journal.pone.0131856 (PMC4526471; doi:10.1371/journal.pone.0131856)
Supplement: S3 File — (DOC) [file pone.0131856.s007.doc]

S3 Appendix - Character list for phylogenetic analysis from Boessenecker and Churchill (2013).

1. Premaxilla: rostral process. 0= absent. 1= present.

2. Anterior narial opening. 0= large, thin margin, and rounded. 1= large, thick margin, and dorsoventrally elliptical. 2= large, thick margin with prenarial shelf.

3. Premaxilla: ascending process along nasal. 0= overlap with nasal long, little contact of nasal and maxilla. 1= short overlap, no contact of ascending premaxilla and frontal. 2= very short overlap.

4. Nasal: posterior end. 0= converging. 1= parallel with transversely straight frontal/maxillary suture. 2= parallel with broad V-shaped frontal/maxillary suture. 3= diverging. 4= penetrating frontal.

5. Palatine fissures (incisive foramina). 0= distinct pair. 1= foramina coalesced. 2= trilobed pits. 3= single pit or reduced.

6. Infraorbital foramen. 0= small. 1= enlarged.

7. Ventral tuberosity of zygomatic root. 0= absent. 1= present.

8. Jugal: anteroventral process. 0= moderate. 1= long, reaching M1. 2= absent.

9. Palate. 0= flat. 1= arched transversely. 2= arched transversely and longitudinally.

10. Maxilla: palatal margins. 0= slightly divergent. 1= parallel. 2= posteriorly very wide.

11. Palatine. 0= small. 1= long and posterolaterally expanded. 2= telescoped and underlying alisphenoid.

12. Pterygoid: hamular process. 0= small and narrow. 1= large and broad.

13. Pterygoid strut. 0= slender. 1= dorsoventrally thin and laterally projected. 2= dorsoventrally thick and laterally broad. 3= laterally thin and rolled.

14. Orbit: fossa muscularis. 0= present. 1= absent.

15. Orbit: antorbital process. 0= small ridge on frontal. 1= prominent on frontal and maxilla. 2= prominent on maxilla only. 3= absent.

16. Frontal: supraorbital process. 0= small bump. 1= absent. 2= large and shelf like.

17. Frontal: interorbital bar. 0= moderate in width, broader than intertemporal region. 1= narrow, parallel sided. 2= very broad and short.

18. Jugal: postorbital process. 0= small. 1= dorsally projecting.

19. Zygomatic process. 0= long and slender. 1= short and slender. 2= dorsoventrally deep. 3= exaggerated.

20. Orbital vacuity. 0= absent. 1= present/anteriorly positioned. 2= present/posteriorly positioned.

21. Orbit: optical foramen and orbitosphenoid. 0= plate-like. 1= funnel shaped.

22. Internal auditory meatus. 0= rounded. 1= bilobed. 2= canals for cranial nerves VII and VIII separated.

23. Epitympanic recess. 0= small. 1= large. 2= very large.

24. Anterior opening of carotid canal. 0= proportionate. 1= retracted.

25. Squamosal fossa on zygomatic root. 0= present. 1= reduced.

26. Glenoid fossa. 0= deep. 1= shallow. 2= laterally shortened.

27. Squamosal: transverse ridge. 0= absent. 1= present.

28. Pseudosylvian sulcus. 0= present, deep. 1= reduced or absent.

29. Bony tentorium. 0= far from petrosal. 1= appressed to petrosal. 2= reduced.

30. Basioccipital. 0= narrow and parallel sided. 1= broad and pentagonal.

31. Posterior lacerate foramen. 0= round. 1= transversely expanded. 2= fissure.

32. Lambdoidal crest. 0= crestlike, posterodorsally directed. 1= flattened, anterodorsally directed.

33. Mastoid process. 0= small. 1= large. 2= very large as widest part of skull. 3= pachyostotic.

34. Paroccipital process. 0= small and separated from mastoid process. 1= enlarged posteriorly but still separate from mastoid process. 2= moderately sized and joined with mastoid process by a continuous ridge. 3= flattened and plate-like.

35. Mandible: fusion of mandibular symphysis. 0= absent. 1= present.

36. Mandible: length of mandibular symphysis. 0=less than 50% of length of horizontal ramus. 1= greater than 50% of length of horizontal ramus.

37. Mandible: genial tuberosity. 0= absent or indistinct. 1= present; developed as small tubercle or process on anterior portion of ramus. 2= present and well developed; extends well below ventral margin of ramus.

38. Mandible: anterior portion of symphyseal region. 0= smooth, compact bone. 1=rugose, vascular bone.

39. Mandible: horizontal ramus. 0= horizontal. 1= upturned.

40. Mandible: mandibular furrow. 0= absent. 1= anterodorsal end of mandibles have a longitudinal furrow anteriorly.

41. Mandible: edentulous mandibular terminus. 0= absent. 1= present.

42. Mandible: posteroventral terminus of mandibular symphysis. 0= same level or anterior to P_1_ or P_2_. 1= posterior to the level of P_2_.

43. Mandible: depth of horizontal ramus. 0= deepest part of horizontal ramus posterior to mandibular symphysis. 1= deepest part of horizontal ramus at posteroventral terminus of symphysis.

44. Mandible: mandibular arch. 0= nearly parallel. 1= sharply divergent.

45. Mandible: ventral border. 0= straight. 1= sinuous.

46. Mandible: enlarged digastric insertion. 0= absent. 1= present.

47. Mandible: mandibular condyle. 0 = at or slightly above level of tooth row. 1 = elevated above tooth row.

48. Mandible: mandibular foramen. 0 = directed anteroventrally. 1 = directed anterodorsally.

49. Mandible: medial shelf of angular process. 0= does not form medial shelf. 1= forms small medial shelf.

50. Mandible: base of coronoid process. 0= narrow (less than 42% of mandible length). 1= broad (greater than or equal to 42% of mandible length).

51. Upper incisors: number. 0= three. 1= two. 2= one. 3= incisors absent.

52. Upper I^3^. 0= moderate. 1= long and slender. 2= premolariform. 3= absent.

53. Lower incisors: number. 0= 2 incisors. 1= 1 incisor. 2= incisors absent.

54. Lower incisors: relative size. 0= lower incisors of equal size, form transverse arcade. 1= lateral incisors greater in size than medial incisors, medial incisor placed posteromedial to lateral incisor.

55. C^1^. 0= proportional. 1= tusk like. 2= tusk like with globular osteodentine.

56. C_1_: size compared to C^1^. 0= nearly equal (100-80%) and caniniform. 1= reduced (75-20%) and premolariform. 2= lower canine absent.

57. C_1_: posterior crista. 0= present. 1= absent.

58. C_1_: longitudinal fluting. 0= absent. 1= present.

59. C_1_: root. 0= oval or circular in cross section. 1= bilobate in cross section or prominent longitudinal labial sulcus.

60. C_1_: orientation. 0= not procumbent and vertically oriented. 1= procumbent.

61. Lower premolars. 0= P_1-4_ present. 1= P_1-3_ present, P_4_ absent. 2= premolars absent.

62. Postcanines: tooth enamel. 0= well developed. 1= thin or patchy. 2= enamel absent in postcanine teeth of adults.

63. Postcanines: crowns. 0= laterally compressed. 1= bulbous.

64. Postcanines: root lobes. 0= root lobes of postcanine teeth of equal or narrower width than crowns. 1= root lobes of postcanine teeth inflated and wider than crowns.

65. Lower postcanines: tooth row length. 0= long (length of tooth row greater than 40% of the mandible length). 1= short (length of tooth row less than or equal to 40% of the mandible length).

66. Lower postcanines: paraconid cusps. 0= present and well developed. 1= absent or reduced.

67. Lower postcanines: lingual cingula. 0= present and smooth. 1= present and rough or crenulated.

68. Lower premolars: hypoconid cusps. 0= present. 1= absent.

69. Lower postcanines: talonid basin. 0= absent. 1= slight concavity or small shelf.

70. Lower postcanines: metaconid. 0= present. 1= reduced. 2= absent.

71. P^1-2^, lingual cingulum. 0= distinct but small. 1= well developed with cuspules. 2= weak and bulbous. 3= P^1-2^ absent.

72. P_2_: rooting. 0= double or bilobate. 1= single.

73. P_3-4_: rooting. 0= double. 1= single.

74. P^3^: roots. 0= double rooted. 1= single and bilobed. 2= single and cylindrical. 3= P3 absent.

75. P^4^, protocone shelf. 0= anteromedially placed. 1= posteromedially placed with small cuspules. 2= reduced or absent.

76. P^4^, roots. 0= triple rooted. 1= double rooted. 2= single rooted. 3= P^4^ absent.

77. M^1^: roots. 0= triple rooted. 1= double rooted. 2= single rooted. 3= M1 absent.

78. M_1_. 0= present. 1= absent.

79. M_1_: rooting. 0= double rooted. 1= single rooted.

80. M^2^: rooting. 0= double rooted. 1= single rooted. 2= M^2^ absent.

81. M_2_. 0= present. 1= absent.

82. Postcanine tooth wear. 0= absent. 1= minor wear on anterior and posterior cusps or apical wear. 2= large wear facets on anterior and posterior edges. 3= heavily worn and polished.

83. Humerus: deltoid tubercle. 0= on pectoral crest. 1= on lateral edge of crest. 2= off crest.

84. Humerus: diameter of distal trochlea. 0= medial lip same diameter (or smaller) as distal capitulum. 1= medial lip diameter greater than distal capitulum.

85. Radius: distal end. 0= unexpanded. 1= expanded, with small radial process. 2= expanded, with large radial process.

86. Metacarpal I: insertion of pollicle extensor. 0= smooth. 1= pit. 2= rugosity.

87. Scapholunar. 0= no pit for magnum. 1= well-formed pit.

88. Astragalus: calcaneal process. 0= absent. 1= present. 2= elongated.

89. Calcaneum: calcaneal tuber. 0= straight. 1= medially prominent.

90. Entocuneiform/mesocuneiform articulation. 0= abutting. 1= overlapping.

91. The spinous process of the axis. 0= axe-like. 1= blade-like.

[New character] Derived conditon, blade-like dorsal margin of the spinous process is seen in the Odobenini, *Valenictus chulavistensis and* *Odobenus rosmarus.* An ancient odobenid *Archaeodobenus akamatsui* also share the derived condition with them. However, another fossil odobenid, *Pseudotaria muramotoi* shows axe-like, curved dorsal margin of the spinous process.
